# A common variant in *AAK1* reduces risk of noise-induced hearing loss

**DOI:** 10.1093/nsr/nwad080

**Published:** 2023-03-20

**Authors:** Qixuan Wang, Xueling Wang, Tao Yang, Lu Yang, Huihui Liu, Yihang Zheng, Guixian Jiang, Hongchao Liu, Chenhui Huang, Juan Chen, Zhentao Wang, Zhaoyan Wang, Wei Zhao, Jiannan Lin, Xuejie Zhang, Junbo Shi, Kun Han, Xingyu Le, Yan Ren, Yun Li, Yingying Hong, Wentao Shi, Dongqi Cui, Minfei Qian, Jun Xu, Xiaofei Zheng, Yunge Gao, Chen Li, James Lin, Zhiwu Huang, Hao Wu

**Affiliations:** Department of Otolaryngology-Head and Neck Surgery, Shanghai Ninth People's Hospital, Shanghai Jiao Tong University School of Medicine, China; Ear Institute, Shanghai Jiao Tong University School of Medicine, China; Shanghai Key Laboratory of Translational Medicine on Ear and Nose Diseases, China; Department of Otolaryngology-Head and Neck Surgery, Shanghai Ninth People's Hospital, Shanghai Jiao Tong University School of Medicine, China; Ear Institute, Shanghai Jiao Tong University School of Medicine, China; Shanghai Key Laboratory of Translational Medicine on Ear and Nose Diseases, China; Biobank, Shanghai Ninth People's Hospital, Shanghai Jiao Tong University School of Medicine, China; Department of Otolaryngology-Head and Neck Surgery, Shanghai Ninth People's Hospital, Shanghai Jiao Tong University School of Medicine, China; Ear Institute, Shanghai Jiao Tong University School of Medicine, China; Shanghai Key Laboratory of Translational Medicine on Ear and Nose Diseases, China; Department of Otolaryngology-Head and Neck Surgery, Shanghai Ninth People's Hospital, Shanghai Jiao Tong University School of Medicine, China; Ear Institute, Shanghai Jiao Tong University School of Medicine, China; Shanghai Key Laboratory of Translational Medicine on Ear and Nose Diseases, China; Department of Otolaryngology-Head and Neck Surgery, Shanghai Ninth People's Hospital, Shanghai Jiao Tong University School of Medicine, China; Ear Institute, Shanghai Jiao Tong University School of Medicine, China; Shanghai Key Laboratory of Translational Medicine on Ear and Nose Diseases, China; Department of Otolaryngology-Head and Neck Surgery, Shanghai Ninth People's Hospital, Shanghai Jiao Tong University School of Medicine, China; Ear Institute, Shanghai Jiao Tong University School of Medicine, China; Shanghai Key Laboratory of Translational Medicine on Ear and Nose Diseases, China; Department of Otolaryngology-Head and Neck Surgery, Shanghai Ninth People's Hospital, Shanghai Jiao Tong University School of Medicine, China; Ear Institute, Shanghai Jiao Tong University School of Medicine, China; Shanghai Key Laboratory of Translational Medicine on Ear and Nose Diseases, China; Department of Otolaryngology-Head and Neck Surgery, Shanghai Ninth People's Hospital, Shanghai Jiao Tong University School of Medicine, China; Ear Institute, Shanghai Jiao Tong University School of Medicine, China; Shanghai Key Laboratory of Translational Medicine on Ear and Nose Diseases, China; Shanghai Institute of Precision Medicine, Shanghai Ninth People's Hospital, Shanghai Jiao Tong University School of Medicine, China; Shanghai Institute of Precision Medicine, Shanghai Ninth People's Hospital, Shanghai Jiao Tong University School of Medicine, China; Department of Otolaryngology-Head and Neck Surgery, Shanghai Ninth People's Hospital, Shanghai Jiao Tong University School of Medicine, China; Ear Institute, Shanghai Jiao Tong University School of Medicine, China; Shanghai Key Laboratory of Translational Medicine on Ear and Nose Diseases, China; Department of Otolaryngology-Head and Neck Surgery, Shanghai Ninth People's Hospital, Shanghai Jiao Tong University School of Medicine, China; Ear Institute, Shanghai Jiao Tong University School of Medicine, China; Shanghai Key Laboratory of Translational Medicine on Ear and Nose Diseases, China; Department of Otolaryngology-Head and Neck Surgery, Shanghai Ninth People's Hospital, Shanghai Jiao Tong University School of Medicine, China; Ear Institute, Shanghai Jiao Tong University School of Medicine, China; Shanghai Key Laboratory of Translational Medicine on Ear and Nose Diseases, China; The Core Laboratory in the Medical Center of Clinical Research, Shanghai Ninth People's Hospital, Shanghai Jiao Tong University School of Medicine, China; Biobank, Shanghai Ninth People's Hospital, Shanghai Jiao Tong University School of Medicine, China; Department of Otolaryngology-Head and Neck Surgery, Shanghai Ninth People's Hospital, Shanghai Jiao Tong University School of Medicine, China; Ear Institute, Shanghai Jiao Tong University School of Medicine, China; Shanghai Key Laboratory of Translational Medicine on Ear and Nose Diseases, China; Department of Otolaryngology-Head and Neck Surgery, Shanghai Ninth People's Hospital, Shanghai Jiao Tong University School of Medicine, China; Ear Institute, Shanghai Jiao Tong University School of Medicine, China; Shanghai Key Laboratory of Translational Medicine on Ear and Nose Diseases, China; Department of Otolaryngology-Head and Neck Surgery, Shanghai Ninth People's Hospital, Shanghai Jiao Tong University School of Medicine, China; Ear Institute, Shanghai Jiao Tong University School of Medicine, China; Shanghai Key Laboratory of Translational Medicine on Ear and Nose Diseases, China; Department of Otolaryngology-Head and Neck Surgery, Shanghai Ninth People's Hospital, Shanghai Jiao Tong University School of Medicine, China; Ear Institute, Shanghai Jiao Tong University School of Medicine, China; Shanghai Key Laboratory of Translational Medicine on Ear and Nose Diseases, China; Department of Otolaryngology-Head and Neck Surgery, Shanghai Ninth People's Hospital, Shanghai Jiao Tong University School of Medicine, China; Ear Institute, Shanghai Jiao Tong University School of Medicine, China; Shanghai Key Laboratory of Translational Medicine on Ear and Nose Diseases, China; Department of Otolaryngology-Head and Neck Surgery, Shanghai Ninth People's Hospital, Shanghai Jiao Tong University School of Medicine, China; Ear Institute, Shanghai Jiao Tong University School of Medicine, China; Shanghai Key Laboratory of Translational Medicine on Ear and Nose Diseases, China; Clinical Research Center, Shanghai Ninth People's Hospital, Shanghai Jiao Tong University School of Medicine, China; Clinical Research Center, Shanghai Ninth People's Hospital, Shanghai Jiao Tong University School of Medicine, China; Department of Otolaryngology-Head and Neck Surgery, Shanghai Ninth People's Hospital, Shanghai Jiao Tong University School of Medicine, China; Ear Institute, Shanghai Jiao Tong University School of Medicine, China; Shanghai Key Laboratory of Translational Medicine on Ear and Nose Diseases, China; Department of Otolaryngology-Head and Neck Surgery, Shanghai Ninth People's Hospital, Shanghai Jiao Tong University School of Medicine, China; Ear Institute, Shanghai Jiao Tong University School of Medicine, China; Shanghai Key Laboratory of Translational Medicine on Ear and Nose Diseases, China; Department of Otolaryngology-Head and Neck Surgery, Shanghai Ninth People's Hospital, Shanghai Jiao Tong University School of Medicine, China; Ear Institute, Shanghai Jiao Tong University School of Medicine, China; Shanghai Key Laboratory of Translational Medicine on Ear and Nose Diseases, China; Department of Otolaryngology-Head and Neck Surgery, Shanghai Ninth People's Hospital, Shanghai Jiao Tong University School of Medicine, China; Ear Institute, Shanghai Jiao Tong University School of Medicine, China; Shanghai Key Laboratory of Translational Medicine on Ear and Nose Diseases, China; Network and Information Center, Shanghai Jiao Tong University, China; Network and Information Center, Shanghai Jiao Tong University, China; Department of Otolaryngology-Head and Neck Surgery, Shanghai Ninth People's Hospital, Shanghai Jiao Tong University School of Medicine, China; Ear Institute, Shanghai Jiao Tong University School of Medicine, China; Shanghai Key Laboratory of Translational Medicine on Ear and Nose Diseases, China; College of Health Science and Technology, Shanghai Jiao Tong University School of Medicine, China; Department of Otolaryngology-Head and Neck Surgery, Shanghai Ninth People's Hospital, Shanghai Jiao Tong University School of Medicine, China; Ear Institute, Shanghai Jiao Tong University School of Medicine, China; Shanghai Key Laboratory of Translational Medicine on Ear and Nose Diseases, China; College of Health Science and Technology, Shanghai Jiao Tong University School of Medicine, China

Noise-induced hearing loss (NIHL) is one of the most common types of hearing loss and contributes to significant morbidity worldwide, as well as being the major cause of age-related hearing loss and tinnitus. The World Health Organization (WHO) estimates that 10% of the world’s population is exposed to sound levels that could potentially cause NIHL [1]. It has been widely accepted that NIHL is a multifactorial genetic disorder with estimated heritability of 36% to 40% [2]. Ideally, identification of the modifying genetic variants would help protect individuals at high risk of NIHL. For decades, many geneticists have searched for genes that either predispose or protect from NIHL. However, the majority of genetic studies for NIHL were conducted in independent known candidate genes [3]. Few genome-wide studies have been implemented to identify novel susceptible variants [4,5], while none of those identified genetic effects have been confirmed functionally. Previous genetic studies of this disorder have been hampered mainly by difficulties in controlling for environmental exposure in order to identify the real NIHL-susceptible cases and resistant controls.

In this study, we obtained a noise exposure history, hearing health questionnaire and pure tone audiograms (PTAs) from 11 070 Chinese Han workers in a shipyard. By audiologic and genetic analysis of this cohort, we showed that a common variant in *AAK1* associates with a lower risk of NIHL. The genetic study comprised two stages: the exome-wide association study (EWAS)-based discovery stage followed by a verification stage (Fig. 1A). Subjects for both stages were taken, via screening, from workers exposed to occupational noise (9850 males and 1220 females, with ages ranging from 18 to 60 years). Among 6367 workers (5785 males and 582 females) who donated 2 ml whole blood samples, a total of 1671 unrelated Chinese Han males were finally included in the genetic studies. For the discovery set, 202 male subjects (101 pairs) with strong NIHL susceptibility and resistance (matched by age, job type and other hearing behavioral covariates, equal or higher noise exposure dose, and better hearing for the resistance group) were initially selected for whole-exome sequencing (WES). One sample from the resistant group was then excluded from the EWAS analysis because of the unqualified genomic DNA. Eventually, the discovery set comprised 201 males, including 101 susceptible and 100 resistant subjects. The ages of the NIHL-susceptible group were matched to that of the resistant group, while the length of noise exposure time and cumulative noise exposure (CNE) dosage of the susceptible group were lower by design (Supplementary Table S1). The NIHL-susceptible group showed typical binaural high-frequency notched hearing loss as the primary sign of NIHL [6], while the resistant group showed normal hearing (Fig. 1B).

**Figure 1.**
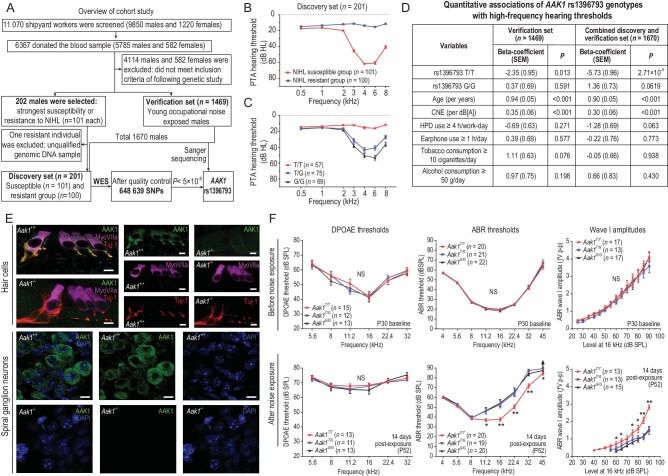
*AAK1* rs1396793 reduces risk of noise-induced hearing loss. (A) Flowchart showing the study design, cohort collection and selecting criteria. (B) The better pure tone audiometric (PTA) hearing thresholds of the NIHL-resistant group than the NIHL-susceptible group. (C) Better PTA thresholds in individuals with the T/T alleles of *AAK1* rs1396793 in comparison with those with the T/G and G/G alleles. Values and error bars reflect mean ± SEM. (D) Quantitative associations of *AAK1* rs1396793 genotypes with high-frequency hearing thresholds. SEM, standard error of mean; CNE, cumulative noise exposure; HPD, hearing protection device. (E) Expression of AAK1 in P1 mouse cochlear hair cells section and spiral ganglion neurons section. The figure shows the extensive expression of AAK1 in hair cells (marked by MyoVIIa), the dendrite of spiral ganglion neurons (marked by Tuj-1) and the cell body of spiral ganglion neurons (marked by DAPI). The specificity of the AAK1 staining is confirmed using the *Aak1*^−/−^ mice as the negative control. Scale bars: 5 μm. (F) Reduced NIHL in the *Aak1* rs1396793-knock-in mice. The figure shows DPOAE thresholds, ABR hearing thresholds and wave I amplitudes of the Aak1 rs1396793-knock-in mice before (P30 baseline) and 14 days after chronic noise exposure (P52). The ABR wave I amplitudes are shown at 16 kHz, one of the frequencies affected most by noise exposure. The up arrow in E indicates no ABR response at the highest stimulus level (90 dB SPL) tested. Statistical differences were analyzed by Kruskal-Wallis analysis of variances (ANOVAs) with Dunn-Bonferroni tests for *post-hoc* comparisons (*P* values in Supplementary Table S3). NS: not significant, ^*^P < 0.05, ^**^P < 0.01. Values and error bars reflect mean ± SEM.

After quality control of the sequencing data, a total of 646 319 single nucleotide polymorphisms (SNPs) were tested for the genetic association with NIHL. The quantile-quantile (Q-Q) plots for each analysis demonstrate a close agreement with the null hypothesis, until the tail of the distribution, where SNPs with chi-squared values <20 become more significant than expected by chance alone (Supplementary Fig. S1). Population stratification with multidimensional scaling was assessed by principal component analysis (PCA) of the SNP genotype, suggesting that subjects in this study have consistent genetic ancestry with the East Asian population in the 1000 Genomes Project (Supplementary Fig. S2). An EWAS of the discovery set detected no candidate variant in genes previously linked to mendelian hereditary deafness. The rs1396793 within the 5^′^ untranslated region (UTR) region of the *AAK1* gene on chromosome 2p13.3 showed the strongest association (*P* = 1.07 × 10^−13^, odds ratio [OR] = 0.13 with 95% confidence interval [95% CI]: 0.08–0.20) with resistance to NIHL (Supplementary Fig. S3). A subsequent permutation test confirmed that the association at rs1396793 was beyond what would be expected by chance (permutation *P* < 0.001). No other genome-wide significant loci (*P* < 5 × 10^−8^) were observed. According to the validated genotype at rs1396793 of the discovery set via Sanger sequencing, subjects carrying the T/T (or A/A) alleles (*n* = 57) had better high-frequency hearing thresholds, suggesting strong resistance to NIHL. In contrast, subjects with the T/G and G/G genotypes showed the high-frequency notched hearing loss characteristic of NIHL (Fig. 1C and Supplementary Table S2).

To verify the association between the *AAK1* rs1396793 variant and NIHL, we subsequently genotyped this variant in 1469 male subjects in the verification set. For quantitative association analysis, the subjects were grouped based on their genotypes (T/T, T/G and G/G alleles). No deviation from the Hardy-Weinberg equilibrium was detected. Their characteristics are shown in Supplementary Table S1. Similar to the results in the discovery set, subjects in the verification set (*n* = 1469) with the T/T alleles at rs1396793 (*n* = 217, 14.8%) are associated with significantly better hearing thresholds at 3, 4, 6 and 8 kHz than those with the G/T (*n* = 718, 48.9%) and G/G (*n* = 534, 36.3%) alleles (Supplementary Fig. S4). By multivariable linear regression analysis, after adjusting for possible confounding factors as a previous study suggested [7] (including age, CNE, hearing protection device [HPD] and earphone use, and tobacco and alcohol consumption), the T/T alleles at rs1396793 in *AAK1* show a significant association with the averaged high-frequency hearing thresholds at 3, 4, 6 and 8 kHz (beta = −2.35, standard error of mean [SEM] = 0.95, *P* = 0.013 for the verification set only; beta = −5.73, SEM = 0.96, *P* = 2.71 × 10^−9^ for the combined discovery and verification set, Fig. 1D).

*AAK1* rs1396793 resides in a highly conservative region in vertebrates (Supplementary Fig. S5) and mouse AAK1 has 90.7% protein similarity to the human counterpart (in the UniProt Knowledgebase). AAK1 is highly expressed in hair cells and spiral ganglion neurons of the mouse cochlea from birth to adulthood, based on our results (Fig. 1E and Supplementary Fig. S6) and the Gene Expression Analysis Resource (gEAR; https://umgear.org). At the age of 1 month, the *Aak1^−^^/^^−^* knock-out mice showed impaired hearing characterized by increased thresholds for distortion product otoacoustic emission (DPOAE) and auditory brainstem response (ABR) and decreased ABR wave I amplitudes, though the overall morphology of the inner ear showed no apparent hair cell loss (Supplementary Fig. S7 and Table S3). The scanning electron microscopy assay showed degeneration of hair bundles for both outer hair cells (OHCs) and inner hair cells (IHCs) in *Aak1^−^^/^^−^* knock-out mice, while the prestin expression in OHCs is mostly normal (Supplementary Fig. S7). These data suggest that AAK1 may have a broad function in OHCs, IHCs and auditory neurons.

The rs1396793-knock-in mice were exposed to bandpass-filtered white noise spanning 2–20 kHz at 106 dB SPL for 2 hours as the acute exposure, and 2 hours per day for 7 days as the chronic exposure. Littermates of all three genotypes had normal hearing at the age of 1 month, but showed variable degrees of permanent hearing impairment 14 days after chronic noise exposure. Strikingly, while the DPOAE threshold elevations were comparable after noise exposure in different genotypes, the *Aak1^T/T^* mice had significantly less ABR threshold elevation and wave I amplitude decrement at higher frequencies than the *Aak1^T/G^* and *Aak1^G/G^* mice (Fig. 1F). The same results can be observed in both male and female mice (Supplementary Fig. S8 and Table S4). Consistent with the auditory electrophysiological findings, quantitative reversed transcription-polymerase chain reaction (qRT-PCR) results showed that the expression of *Aak1* in total cochlea, OHCs and IHCs of the *Aak1^T/T^* mice was temporarily activated at 1 day post acute noise exposure, but not in the *Aak1^G/G^* mice (Supplementary Fig. S9). These data support that the rs1396793 variant in *Aak1* protects from NIHL in mice.

This is to our knowledge the first report to implicate *AAK1* in NIHL or auditory function in general. *AAK1* encodes a serine/threonine kinase of the Ark1/Prk1 family. In neuronal cells, AAK1 is highly expressed at the presynaptic terminals and regulates clathrin-mediated endocytosis (CME) by phosphorylating the μ subunit of adaptor protein complex 2 (AP-2μ), which probably plays roles in autophagy, dendrite arborization, synapse formation, etc. [8,9]. A current proteomic study of synaptic vesicles (SVs) in the mammalian brain further characterized AAK1 as an abundant and SV-resident kinase essential for SV recycling and high-frequency neurotransmission [10]. Though the auditory function of AAK1 remains elusive, recent studies showed that CME is required for SV reformation and endocytosis in cochlear hair cells, and AP-2μ plays a critical role in cochlear sound encoding. Conditional knock-out of *AP-2μ* slows the rapid exocytosis and SV reloading at the release sites of the mouse IHCs and leads to profound hearing impairment [11]. Thus, it is conceivable that AAK1 is involved in NIHL, probably by controlling the phosphorylation status of AP-2μ in the cochlea.

Our studies indeed support the theory that AAK1 is at least moderately required for proper auditory function, as the *Aak1* knock-out mice have elevated ABR hearing thresholds. The T/T alleles of rs1396793 knock-in mice were associated with better ABR wave I amplitudes at high frequencies (Fig. 1F), consistent with the hypothesized cochlear synaptic function related to the AAK1: AP-2μ pathway. On the other hand, despite the fact that DPOAE were markedly impaired in all genotype groups of the knock-in mice after noise exposure, we could not exclude the potential role of rs1396793 in the protection of OHC function. Further studies are required to clarify the mechanism underlying the protective role of *AAK1* rs1396793 in NIHL.

The main limitation of this study is the relatively small sample size of the discovery set, which is due to the rigorous selection criteria employed. Nevertheless, we believe that our well-matched, deeply phenotypic subjects with strong susceptibility and resistance to NIHL are unprecedented and no small task in studies of NIHL. This likely improved the statistical power of the analysis [12]. Moreover, rs1396793 is a common variant in several ethnic populations, including East Asians, South Asians and Africans (with minor allele frequencies [MAFs] of 0.372, 0.164 and 0.193, respectively). Ethnic variability clearly exists [13] as MAFs range from 0.035 to 0.06 for Europeans. Although we have determined the association between the genotype of T/T alleles at rs1396793 with NIHL in an East Asian verification, future validation of this variant may be more difficult in some ethnic populations. Also, due to the potential influence of sex on NIHL [7] and the low percentage of female workers among the subjects, our human studies focused on male subjects only. Though our mouse studies showed little difference between male and female mice, this remains to be verified in female human subjects.

In summary, we have identified that variant rs1396793 in the 5^′^UTR of *AAK1* is associated with a reduced risk of NIHL. These results suggest that regulatory patterns of genetic and environmental interactions exist in complex diseases. Genetic screening of this common variant may present an effective, cost-efficient strategy for prevention and intervention with regard to NIHL. AAK1, which plays a key role in cochlear CME, is a promising therapeutic target for the future. Further studies, including validation in additional populations, are required.

## Supplementary Material

nwad080_Supplemental_File
