# Supplementary material for: A common variant in AAK1 reduces risk of noise-induced hearing loss
Source: Natl Sci Rev. 2023 Mar 20;10(7):nwad080. doi: 10.1093/nsr/nwad080 (PMC10401317; doi:10.1093/nsr/nwad080)
Supplement: nwad080_Supplemental_File [file nwad080_supplemental_file.docx]

**Supplementary Data** **for**

**A common variant in *AAK1* reduces risk of noise-induced hearing loss**

**Authors:** Qixuan Wang,^1,2,3,#^ Xueling Wang,^1,2,3,4,#^ Tao Yang,^1,2,3^ Lu Yang,^1,2,3^ Huihui Liu,^1,2,3^ Yihang Zheng, ^1,2,3^ Guixian Jiang, ^1,2,3^ Hongchao Liu,^1,2,3^ Chenhui Huang,^5^Juan Chen,^5^ Zhentao Wang,^1,2,3^ Zhaoyan Wang,^1,2,3^ Wei Zhao,^1,2,3^ Jiannan Lin,^6^ Xuejie Zhang,^4^ Junbo Shi,^1,2,3^ Kun Han,^1,2,3^ Xingyu Le,^1,2,3^ Yan Ren,^1,2,3^ Yun Li,^1,2,3^ Yingying Hong,^1,2,3^ Wentao Shi,^7^ Dongqi Cui,^7^ Minfei Qian,^1,2,3^ Jun Xu,^1,2,3^ Xiaofei Zheng,^1,2,3^ Yunge Gao,^1,2,3^ Chen Li,^8^ James Lin,^8^ Zhiwu Huang (黄治物),^1,2,3,9^* and Hao Wu (吴皓)^1,2,3,9^*

**Affiliations:**

^1^ Department of Otolaryngology-Head and Neck Surgery, Shanghai Ninth People’s Hospital, Shanghai Jiao Tong University School of Medicine, Shanghai 200011, China;

^2^ Ear Institute, Shanghai Jiao Tong University School of Medicine, Shanghai 200025, China;

^3^ Shanghai Key Laboratory of Translational Medicine on Ear and Nose Diseases, Shanghai 200125, China;

^4^ Biobank, Shanghai Ninth People’s Hospital, Shanghai Jiao Tong University School of Medicine, Shanghai 200011, China;

^5^ Shanghai Institute of Precision Medicine, Shanghai Ninth People’s Hospital, Shanghai Jiao Tong University School of Medicine, Shanghai 200011, China;

^6^ The Core Laboratory in Medical Center of Clinical Research, Shanghai Ninth People’s Hospital, Shanghai Jiao Tong University School of Medicine, Shanghai 200011, China;

^7^ Clinical Research Center, Shanghai Ninth People’s Hospital, Shanghai Jiao Tong University School of Medicine, Shanghai, 200011, China;

^8^ Network and Information Center, Shanghai Jiao Tong University, Shanghai 200240, China.

^9^ College of Health Science and Technology, Shanghai Jiao Tong University School of Medicine, Shanghai 200000, China.

^#^ These authors contributed equally to this work.

*** Corresponding Authors:** Hao Wu (Email: [wuhao@shsmu.edu.cn](mailto:wuhao@shsmu.edu.cn)), Zhiwu Huang (Email: huangzw86@163.com).

**METHODS**

**Subjects and study design**

Subjects were recruited between August 2017 and June 2018 from a shipyard in Shanghai, China. For all subjects, hearing thresholds at frequencies 0.5-8 kHz were determined by air-conduction pure tone audiometry (PTA). The noise exposure dosage was estimated using the cumulative noise exposure (CNE)[1, 2], which was calculated according to the equivalent A-weighted sound pressure level of continuous 8 hours in work-day (*L_Aeq,8h_*) for different job types and working-length for each subject. Hearing behavioral covariates, including hearing protection device (HPD), earphone usage, tobacco and alcohol consumption, were recorded through questionnaires.

The general inclusion criteria for genetic studies were the following: (1) Aged 18-50 years; (2) Over one-year occupational noise exposure with stable job types, (3) No history of otological injuries or disorders, (4) No history of ototoxic drug uses, tobacco or alcohol consumption, (5) No family history of hearing loss, (6) No perforation of tympanic membrane or abnormal tympanogram, and (7) Averaged low-frequency (0.5, 1 and 2 kHz) hearing thresholds of less than 50 dB HL in either ear. Sex and race were self-reported. Only young males were included in the genetic study to exclude the possible sex and aging influence on noise-induced hearing loss (NIHL)[3-5]. Characteristics of all subjects are shown in Table S1, which have been confirmed by follow-up inquiry.

**Collection of questionnaires, noise exposure level, and auditory data**

We designed a hearing health questionnaire to obtain the following information: (1) Demographic characteristics including biological sex, age, race and other relevant information, (2) Working environment and occupational noise exposure time-length, (3) Non-occupational sound exposure such as usages of personal earphones (at least 1 hours per day as “yes”) and HPD (at least 4 hours per day as "yes"). (4) Individual behavior habits including tobacco (at least 10 cigarettes per day was marked as "yes") and alcohol consumption (at least 50 g of alcohol per day was marked as "yes"). The questionnaire had been pre-tested in 100 workers through face-to-face interview with practicing investigators, ensuring that all workers can understand and complete the questionnaire within acceptable time.

We assessed the noise exposure level in six types of representative working environment in the shipyard (Table S5). A free field acoustimeter (type AWA6228+, Hangzhou Aihua) was used to measure the equivalent A-weighted sound pressure level of continuous 8 hours (*L_Aeq,8h_*, from 8:00 am. to 4:00 pm.) in working days. CNE was used to estimate the cumulative effects of noise exposure for each subject. Since all subjects included in this study had stable job status, CNE can be calculated using the following formula:

CNE = L_Aeq,8h_ + 10logT.

Where L_Aeq,8h_ is the job-specific noise exposure level and T is the occupational noise exposure time-length in years.

Air-conduction PTA thresholds at frequencies of 0.5,1,2,3, 4, 6 and 8 kHz was measured in 5-dB step by certified audiological technician using the diagnostic audiometer (Madsen Xeta, Otometrics) with TDH-39P headphones in a silent soundproof booth with background noise below 25 dB(A), in accordance with the ISO 8253-1:2010 standard. All subjects would not be exposed to occupational noise within 16 hours before hearing test.

**Exome-wide association study (EWAS) of the discovery set**

For the discovery set, two groups (n = 101 each) of subjects with strong resistance or susceptibility to NIHL were selected from those 1,671 workers. The resistant group was defined as those who had higher noise exposure levels (working length of more than 3 years, and CNE ≥ 94 dB [A]) but normal PTA (lower than 25 dB HL) at any frequencies between 0.5 and 8 kHz. The susceptible subjects were defined and matched with each resistant subject based on the following criteria: (1) Age differences less than 3 years, (2) Same job types, (3) Shorter noise exposure time-length and lower CNE, (4) Moderate-to-severe, high-frequency hearing loss (average hearing thresholds of at least 35 dB HL at frequencies 3, 4, 6 and 8 kHz) with binaural high-frequency audiometric notch[6].

Genomic DNA was extracted from blood using the QIAamp DNA Blood Mini Kit (Qiagen, 51106). Whole-exome target capture of the discovery set was performed by Agilent SureSelect V6 Kit-LT and 150 bp paired-end sequencing reads on the Illumina platform to an average depth of about 120×. Whole-exome sequencing (WES) data was analyzed according to standardized Genome Analysis Tool Kit (GATK) 3.6 pipeline[7, 8] (see details in the supplement**).**

**Bioinformatics**

The raw data was aligned to the Genome Reference Consortium Human Build 37 (GRCh37/hg19) using the Burrows Wheeler Alignment (version 0.7.13) MEM algorithm.[9] Duplication was marked by Picard Markduplicates (version 2.2.4). Base Quality Score Recalibration (BQSR) was performed using the GATK tools (version 3.6.0). SNPs and indels were called by HaplotypeCaller[10] with interval lists specific to the exome enrichment kit. gVCF files were combined by CombineGVCFs. Genotyping was performed using the genotypeGVCF tool implemented in GATK. The cohort callset was filtered with GATK VariantRecalibrator and ApplyVQSR. Annotation of the VCF files was performed by a customized version of ANNOVAR.[11] The allele frequencies of all variants were compared to the Genome Aggregation Database.[12] According to the standard GWAS Quality Control measures,[13] the genotypic association analysis was performed using the chi-square test and permutation test in the software package PLINK (version 1.9).[14] Predicted function of variants on transcription factor binding profiles at the whole genome scale were based on Encyclopedia of DNA Elements (ENCODE) Database.[15]

**Quantitative association analysis of the verification set**

For the verification set, a total of 1,469 subjects were selected from the rest of the 1,671 male participants and genotyped for the rs1396793 variant in *AAK1* (GenBank: NM_014911.4). Sanger sequencing was performed using the BigDye Mix kit (Applied Biosystems) on the 3730XL DNA analyzer (Applied Biosystems) and analyzed by the Sequencher software (version 5.1). Primers for polymerase chain reaction (PCR) were listed in Supplementary Table S6.

**Overview of functional studies in the mouse models**

To functionally assess the role of *Aak1* and the rs1396793 variant in NIHL, *Aak1* knock-out (*Aak1^-/-^*) mice and rs1396793-knock-in (*Aak1^T/T^*) mice of the C57BL/6 background were generated using the CRISPR/Cas9 system[16] (Supplementary Fig. S10). The mice were exposed to the bandpass-filtered white noise spanning 2-20 kHz at 106 dB SPL for two hours as the acute exposure, and two hours per day for seven days as the chronic exposure[17]. All mice compared in noise exposure experiments were littermates in both sexes. Auditory functions were measured by distortion product otoacoustic emission (DPOAE) and auditory brainstem response (ABR) tests[18]. Cochlear expression levels of *Aak1* mRNA were analyzed by quantitative reversed transcription-PCR (qRT-PCR). Spatial expression pattern of AAK1 in mouse cochlea was analyzed by immunofluorescence.

**Generation and genotyping of the *Aak1* knock-out and rs1396793-knock-in mice**

*Aak1* is well conserved between humans and mouse including the nucleotide position of the rs1396793 variant (chromosome 6: 86849933 in the mouse genome GRCm38/mm10). The schematic strategies for both mutant mice were shown in Supplementary Fig. S4A and S4B. In the *Aak1* knock-out (*Aak1^-/-^*) mice, a c.769_770delAA dinucleotide deletion in exon2 of *Aak1* (NM_001040106.3) were introduced, resulting a truncated protein with premature stop after 80 amino acids from the N-terminus. In the *Aak1* rs1396793-knock-in (*Aak1^T/T^*) mice, a single G>T nucleotide change was placed at the chromosome 6: 86849933 position corresponding to the rs1396793 variant in humans. Both mutant mice were generated via the CRISPR/Cas9 system^17^. For the *Aak1* knock-in mice, sgRNA and DNA donor was transcribed *in vitro*. The Cas9 mRNA, sgRNA and DNA donor were then co-injected into the fertilized eggs of C57BL/6 mice. For the knock-out mice, sgRNA transcribed *in vitro* were co-injected with Cas9 mRNA into the fertilized eggs. The fertilized eggs were transplanted to obtain positive F0 mice that were confirmed by sequencing. A stable F1 generation mouse model was obtained by mating positive F0 mice with C57BL/6 mice. Sequences of the sgRNA and DNA donor were shown in Table S6. Genomic DNA of the mice was extracted from the tail or toe using the Qiagen DNeasy Blood and Tissue kit (Qiagen, 69506). Genotyping was performed by PCR amplification and Sanger sequencing. The sequences of the primers were shown in Table S6. Genotyping results (chromatogram) of the knock-out and knock-in mice were shown in Supplementary Fig. S4C and S4D.

**Handling of the mice**

All experimental procedures involving mice were in accordance with the 3R (Replacement, Reduction and Refinement) principles of animal welfare and approved by the Animal Ethics Committee of the Ninth People's Hospital affiliated to Shanghai Jiao Tong University School of Medicine. Mice were housed in a pathogen-free animal facility accredited by Association for Assessment and Accreditation of Laboratory Animal Care International. Mice were maintained under the 12/12 hours of light and dark cycle at 23°C ± 2°C with adequate water and food supply. Boiled egg yolk was fed to the nursing mother of the *Aak1^-/-^* pups. During the period (several weeks) of audiological experiments, mice were transferred into a standard, acoustically quiet animal facility.

**Noise exposure procedures**

Male and female mice at one month of age were both used for the noise exposure experiments. Bandpass-filtered white noise spanning 2-20 kHz was generated using the Matlab software (version 2007b). The acute noise exposure was set at the intensity of 106 dB SPL for 2 hours, while the chronic noise exposure was set as repetition of the acute exposure consecutively for seven days. Acoustimeter (type AWA6228+, Hangzhou Aihua) was used to calibrate the sound stimuli that were presented by an amplifier and loud speaker (Yamaha). The loud speaker was placed 30 cm away from an internally separated wire cage for the exposed mice in a reverberation room. The acute noise exposure stimulation was used to induce the cochlear mRNA expression level changes, while the chronic noise exposure procedure was used to detect the alteration of auditory tests.

**Auditory tests**

ABR and DPOAE were tested in a silent soundproof room. Mice were anesthetized by intraperitoneal injection of a mixture of ketamine (100 mg/kg) and levomepromazin (5 mg/kg) with body temperature maintained at 37°C using an isothermal heating pad (Harvard Apparatus). For ABR tests, Calibration of tone bursts in the 4-45 kHz range was performed using BioSigRZ’s calibration tools and microphone (Tucker Davies Technology) before the test. Needle electrodes were inserted beneath the skin at the vertex for recording, the ipsilateral mastoid for reference and the hind hip for ground. The 4 ms tone bursts (1-2-1-ms ramp, 20/s) at frequencies of 4, 5.6, 8, 11.2, 16, 22.4, 32 and 45kHz were generated starting from 90 dB SPL and downwards in 5-dB step, using the RZ6 auditory processor with MF1 free-field speaker (Tucker Davies Technology). Averaged 400 response waveforms at each frequency and level were recorded by BioSigRZ software. The ABR threshold was defined as the intensity at minimal visual inspection of the stacked waveforms. For chronic noise exposure, ABR thresholds of the mice in different genotypes were recorded at the unexposed baseline, then 14 days post-exposure. For DPOAE tests, the right external auditory meatus was coupled to a small ER10B+ microphone (Etymotic Research). Two EC1 electrostatic speakers were used to generate equal intensity primary tones (f_1_ and f_2_, with f_2_/f_1_ = 1.2) by the Tucker Davis Technologies system. Distortion-product data at the frequency 2f_1_-f_2_ was collected every 21ms and averaged 512 times in response to center frequencies at 5.6, 8, 11.2, 16, 22.4 and 32 kHz presented from 20 to 80 dB SPL (increasing in 5 dB steps). The DPOAE threshold was defined as the point where the distortion-product can no longer be detected from the noise of the microphone.

**Immunofluorescence studies**

Mouse cochleae were dissected rapidly and fixed in 4% paraformaldehyde (vol/vol) in phosphate-buffered saline (PBS) overnight in 4℃ and were then decalcified completely in 0.12 M ethylene diamine tetraacetic acid (EDTA). Cochleae were then microdissected into pieces or embedded in OCT and sectioned with a cryostat at 14μm after cryoprotecting in 30% sucrose (wt/vol) for 12 hours. The sample was pre-incubated in blocking solution (5% donkey serum (vol/vol), 5% goat serum (vol/vol), 0.3% Triton X-100 (vol/vol) in PBS, pH 7.4) for 1 hour at room temperature, next then incubated with primary antibodies at 4℃ overnight. For primary antibodies, we used rabbit anti-AAK1 (Sigma-Aldrich, HPA020289, 1:100), rabbit anti-SLC26A5 (Invitrogen, PA5-103158, 1:500), mouse IgG1 anti-myosin VIIa (Developmental Studies Hybridoma Bank, AB_2282417, 1:50), and mouse IgG2a anti- Tubulin Beta 3 (BioLegend, 657402, 1:500). After washing in PBS, the slides were incubated with secondary antibodies including Goat anti-Mouse IgG1 Alexa Fluor 633 (Invitrogen, A-21126, 1:200), Goat anti-Mouse IgG2a Alexa Fluor 555 (Invitrogen, A-21137, 1:200) and Donkey anti-Rabbit IgG Alexa Fluor 488 (Invitrogen, A-21206, 1:500) at room temperature for 2 hours and then washed three times (10 minutes each) with PBS. The slides were mounted using ProLong™ Gold Antifade Mountant with DAPI (Invitrogen, P36931). Images were acquired and processed using a Zeiss 880 Confocal Laser Microscope System with 63× glycerin-immersion lens, with or without digital zoom.

**Scanning electron microscopy (SEM)**

Mouse cochleae were fixed in in 2.5% glutaraldehyde in PBS at 4 °C overnight and decalcified with 0.5 M EDTA at room temperature for 12 hours, and the organ of Corti was dissected into apical, middle, and basal regions, then rinsed in 1 × PBS three times and post-fixed with 1% osmium tetroxide at room temperature for 1 hour. After complete rinse with 1 × PBS three times, the specimens were treated with 2% tannic acid (V900190-100G, Sigma) and dehydrated through the graded ethanol series for 10 minutes at 30%, 50%, 70%, 90%, and 100% at 4 °C. Then the specimens were critical point dried (Leica EM CPD300) and coated with 8 nm gold (Leica EM ACE200 Vacuum Coater) and observed with Aquilos Cryo-FIB (Thermo Scientific).

**Real-time qRT-PCR**

Mouse cochleae were rapidly removed into RNase-free dish with RNAlater Stabilization Solution (Thermo Scientific, AM7020) and dissected. The total RNA was extracted from organ of Corti using a MiniBEST Universal RNA Extraction Kit (Takara, 9767). Individual OHCs and IHCs were collected from the apical coils of freshly dissected organs of Corti for single cell quantitative PCR (qPCR) as previous study described[19]. Reverse transcription was performed using a PrimeScript RT Master Mix (Takara, RR036A) following the manufacturer's instructions. Real-time qPCR was performed on the LightCycler 480 System (Roche) using the TB Green Premix Ex Taq II (Takara, RR820B). Ct values and levels of *Aak1* mRNA in mouse cochlea were calculated by normalizing to β-actin. Primers detecting *Aak1* and the referenced *β-actin* (for total cochlea), *Slc26a4* (for OHCs) and *Otof* (for IHCs) mRNA were provided in Table S6. For acute noise exposure, cochlear *Aak1* mRNA levels of the mice in different genotypes were analyzed at the unexposed baseline, then 1 day and 3 days post-exposure.

**Statistical analysis**

In the discovery set, unpaired Student’s *t* test or the Mann-Whitney *U* test was applied to compare differences in hearing thresholds. Variants with *P* values lower than 5×10^-8^ in the EWAS were considered as candidates. Post hoc multivariable linear regression analysis was performed with subjects in both the discovery and the verification sets pooled into a combined analysis. Variants were tested for deviations from Hardy-Weinberg disequilibrium using the chi-square test. Kruskal-Wallis ANOVA was used to compare differences in continuous variables among groups with a Dunn-Bonferroni test for post hoc comparisons. One-sample *t* test was used to analyze the difference of the relative mRNA levels. We did not use any data imputation methods as there was no missing data in analyses. Categorical variables are presented as percentages (%), continuous variables are expressed as means (SD) and tested of normality using kurtosis and skewness coefficients. Statistical analyses were performed using the IBM SPSS software (version 25.0) and a two-tailed *P* value of less than 0.05 was considered statistical significance except for the EWAS (as described above).

**Ethics approval**

This study in humans followed the Strengthening the Reporting of Genetic Association Studies (STREGA) guideline and was approved by the Ethics Committee of the Ninth People's Hospital affiliated to Shanghai Jiao Tong University School of Medicine (2017-310-t230). All subjects signed the written informed consent to participate, in adherence to the Declaration of Helsinki. All animal experimental procedures involving mice were approved by the Animal Ethics Committee of the Ninth People's Hospital affiliated to Shanghai Jiao Tong University School of Medicine (SH9H-2020-A606-1).

**Data availability**

All WES data were deposited to the Research Big Data Platform (http://bio.jcloud.sjtu.edu.cn/) of Network and Information Center, Shanghai Jiao Tong University, under dataset number GDSJ2019010801.The data deposited and made public is compliant with the regulations of Ministry of Science and Technology of the People's Republic of China. Relevant data that are included with this study are available from corresponding author upon reasonable request.

**Author Contributions**

Q.W. and Xueling Wang designed the experiments, analyzed data, and wrote the manuscript. T. Y. overviewed the data analyses and wrote the manuscript. C. H. and J. C. designed and generated the mutant mouse models. L. Y., Xu Wu, J. S., K. H., Y. R., Y. L. and D. C. collected, checked and analyzed the cohort data. Zhentao Wang, Zhaoyan Wang, W. Z. and X. L. collected blood samples of the cohort. Y.Z., Y. H. and M. Q. designed and performed the noise exposure experiments in mice and analyzed data. G.J., Huihui Liu, Hongchao Liu, Xuejie Zhang, J. X., Xiaofei Zheng and Y.G. designed and performed the mouse cochlear molecular expression and staining experiments, and analyzed data. Jiannan Lin, C.L. and James Lin analyzed the WES data. W. S. performed and checked the statistical analyses. Z. H. and H. W. designed and supervised this research, and wrote the manuscript. All authors edited the manuscript.

**Acknowledgements**

We thank all subjects for their participation and cooperation; Jiangnan Shipyard and the Worker's Hospital for their administrative support; Ms. Honnora Pan from Shanghai Zhongyi Precision Medical Technology Co., Ltd. for contributing to participant ascertainment; Professor Yong Cai, PhD (School of Public Health, Shanghai Jiao Tong University School of Medicine) and his research team for suggestions on statistical analysis.

**SUPPLEMENTARY FIGURES**


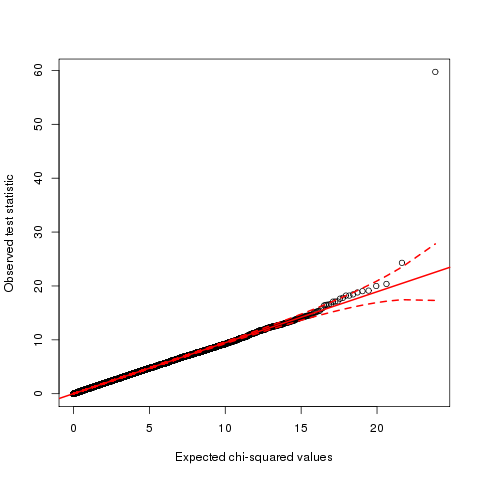


**Figure S1. Quantile-quantile (Q-Q) plots of the discovery set.** The observed (y axis) and the expected (x axis) chi-squared value is plotted for each SNP (dot), and the red solid line indicates the null hypothesis of no true association. Deviation from the expected chi-squared distribution is evident only in the tail area, suggesting that population stratification was adequately controlled.


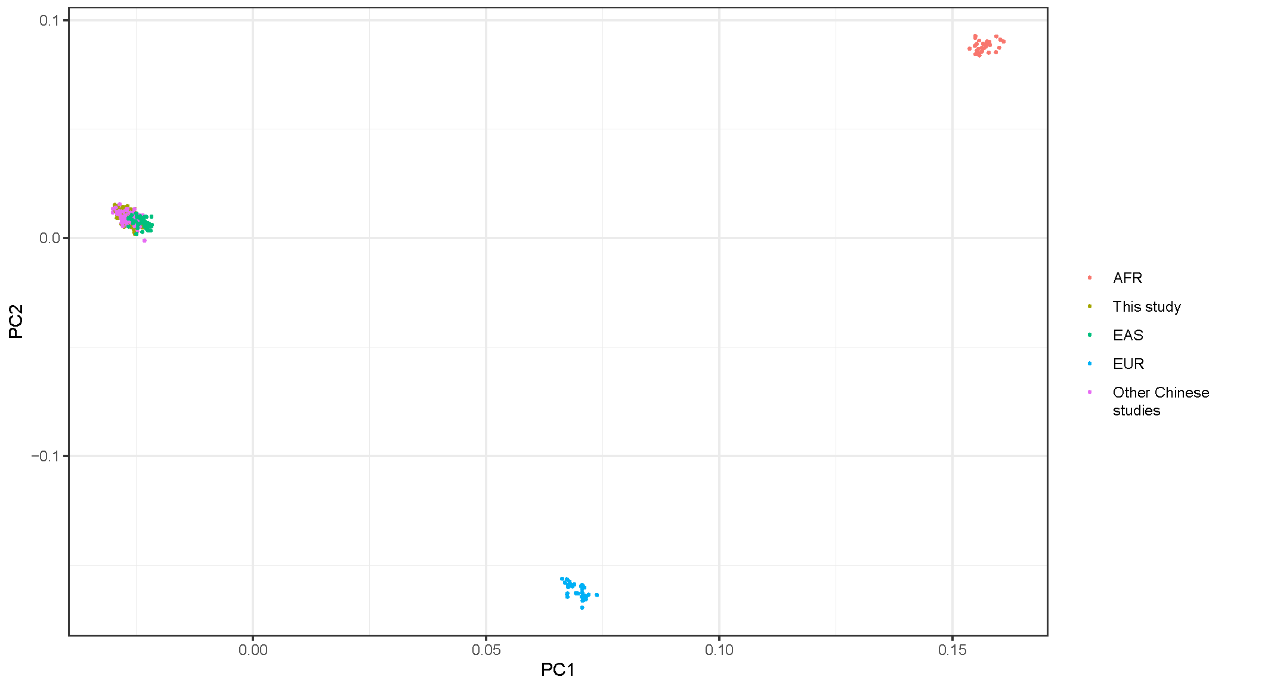


**Figure S2. Multidimensional scaling (MDS) plot in the discovery set.** Genetic ancestry was assessed by principal component analysis (PCA) of SNP genotype. PCA of a combined data including the discovery set in this study, the 1000 Genomes and other Chinese studies provided by Berry Genomics Corporation (Beijing, China). Our samples (in brown) showed the similar genetic structure with the East Asian Ancestry (EAS; in green) population in the 1000 Genomes and other Chinese population (in purple), which are very different from the African Ancestry (AFR; in pink) and European Ancestry (EUR; in blue) population in the 1000 Genomes.


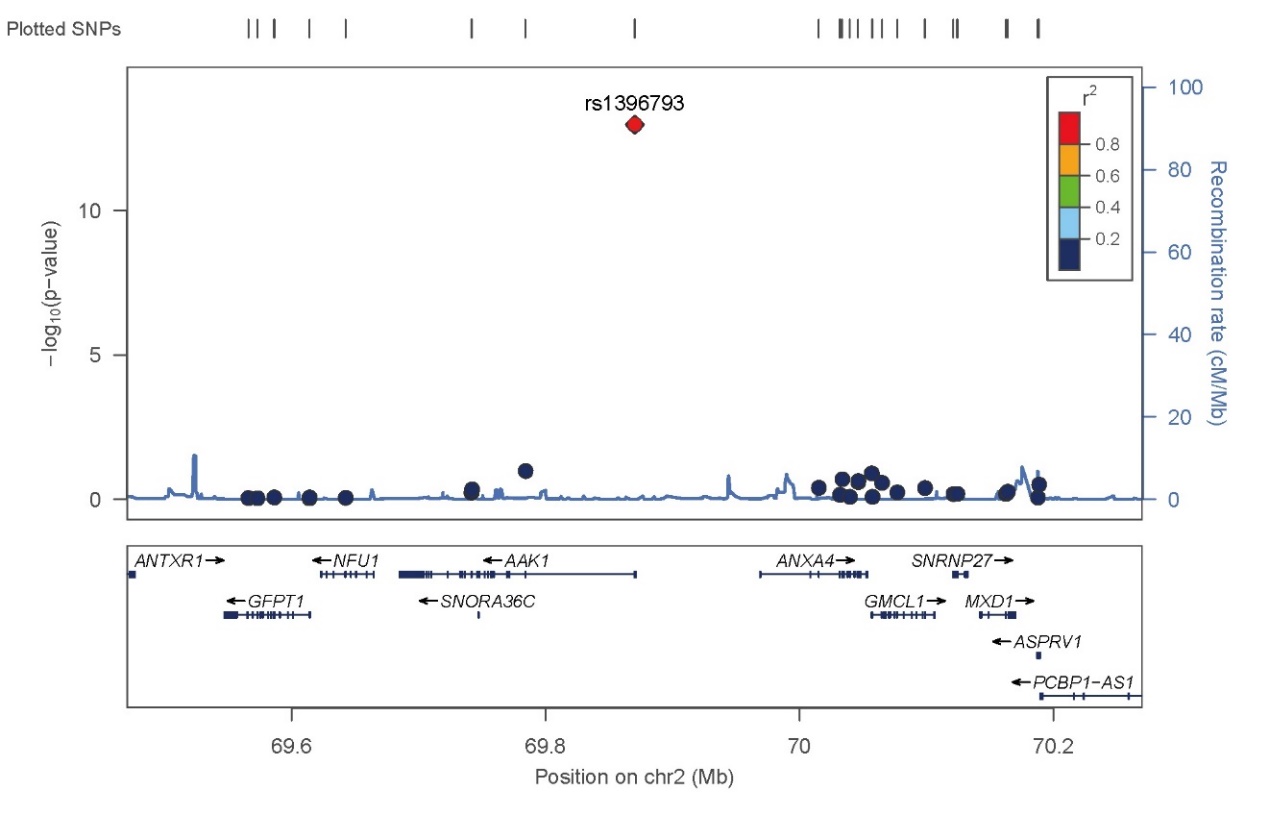


**Figure S3. Loci with possible association to NIHL around rs1396793 in discovery set.** The vertical axis represents statistical significance of the association, measured by the negative log10 of the *P* values, between NIHL and the variants (grey and red dots arranged by their chromosomal location on the horizontal axis). rs1396793 in *AAK1* is labeled in red.


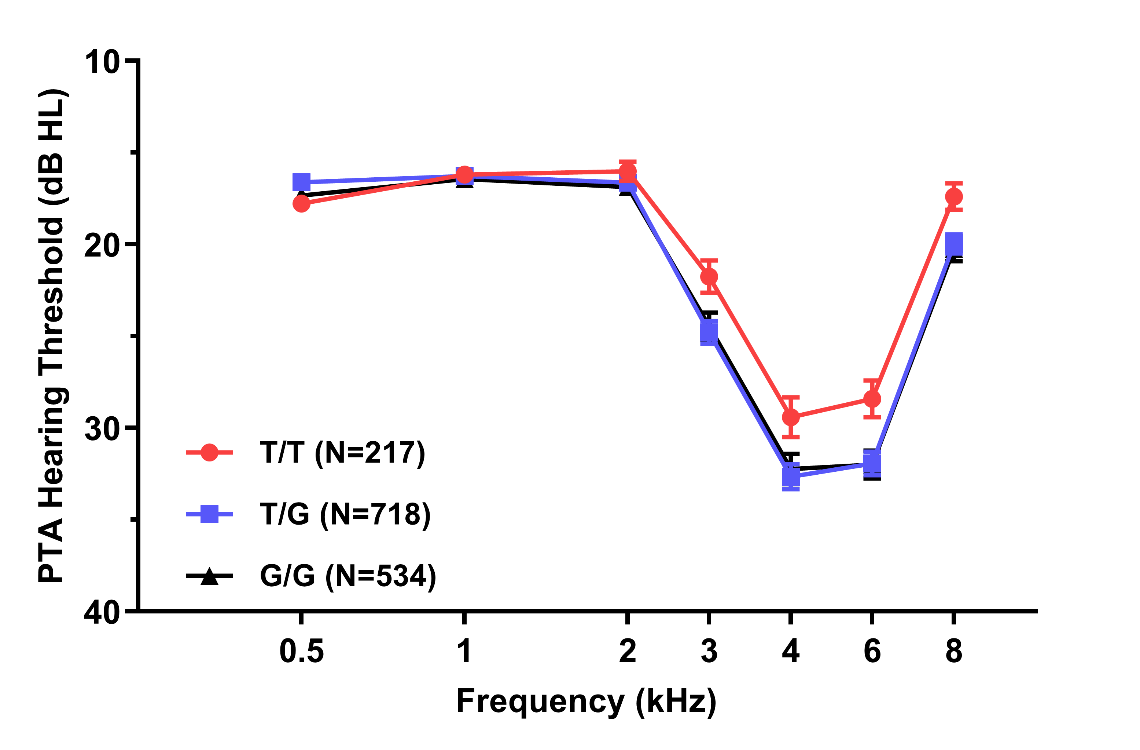


**Figure S4. Reduced risk of NIHL in individuals with T/T alleles of rs1396793 in the verification set (n = 1,469).** Better pure tone audiometric (PTA) thresholds at frequencies 3 - 8 kHz in individuals with the T/T alleles of *AAK1* rs1396793 (n = 217, red) in comparison with those with the T/G (n = 718, blue) and G/G (n = 534, black) alleles. Values and error bars reflect mean ± s.e.m..


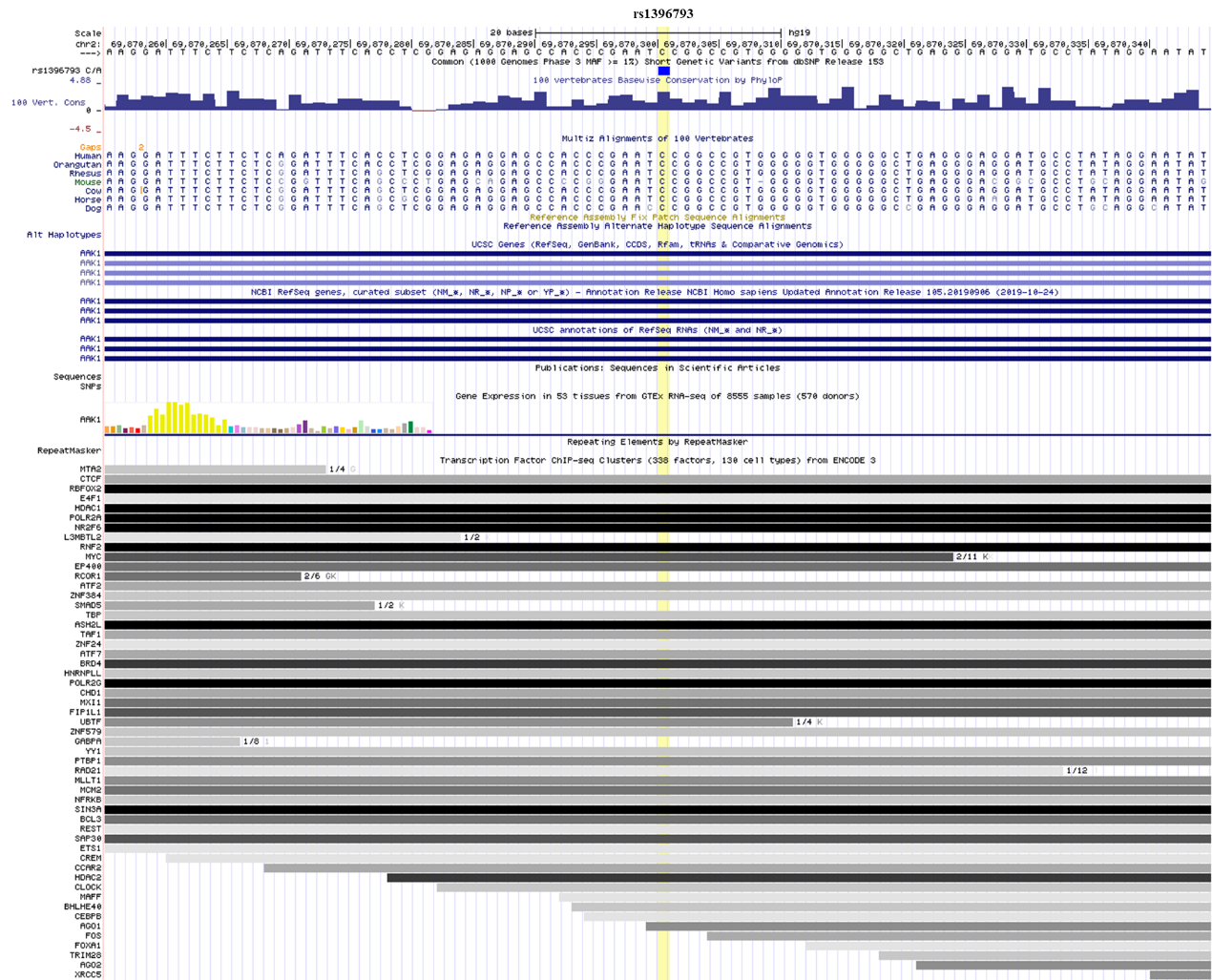


**Figure S5. Conservation of and predicted transcription factor binding of the 5’UTR region of *AAK1* containing rs1396793.** Snapshot from the UCSC Genome Browser database (http://genome.ucsc.edu/) illustrating the genomic region containing rs1396793, which is located in a highly conserved (shown by Multiz alignments in Multivariable vertebrate species) 5'UTR of *AAK1*. Transcription factor CHIP-seq clusters from the ENCODE database are shown in grey bars.


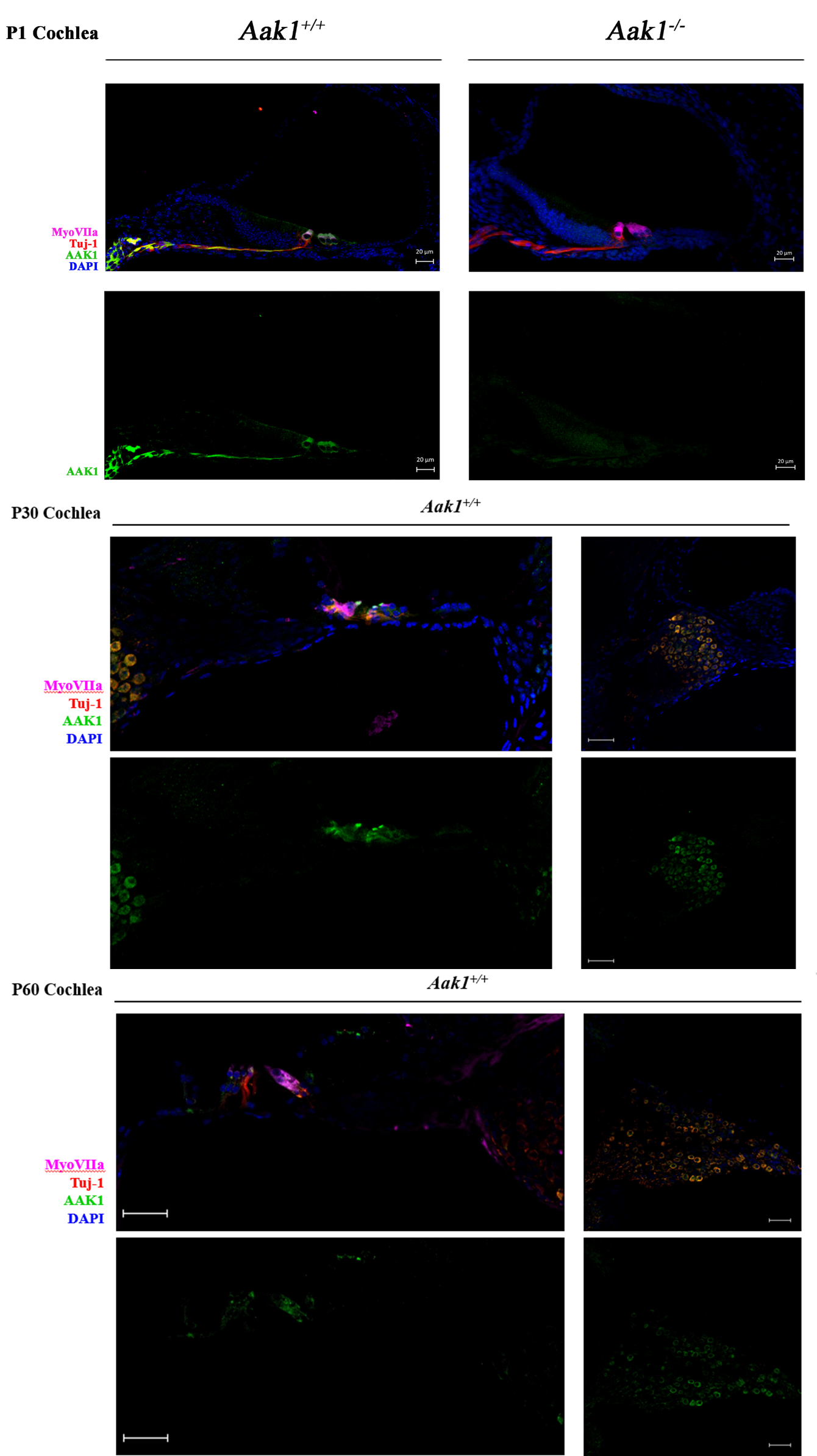


**Figure S6. Expression of AAK1 in the mouse cochlea at different ages.** Immunohistochemistry of P1, P30 and P60 mouse cochlear sections for hair cells and spiral ganglion neurons. Note the extensive expression of AAK1 (green) in hair cells (marked by MyoVIIa, purple) and the dendrite of spiral ganglion neurons (marked by Tuj-1, red), and the expression of AAK1 (green) in the cell body of spiral ganglion neurons (marked by DAPI blue). The specificity of the AAK1 staining is confirmed using the *Aak1^-/-^* mice as the negative control. Scale bars: 20 μm.


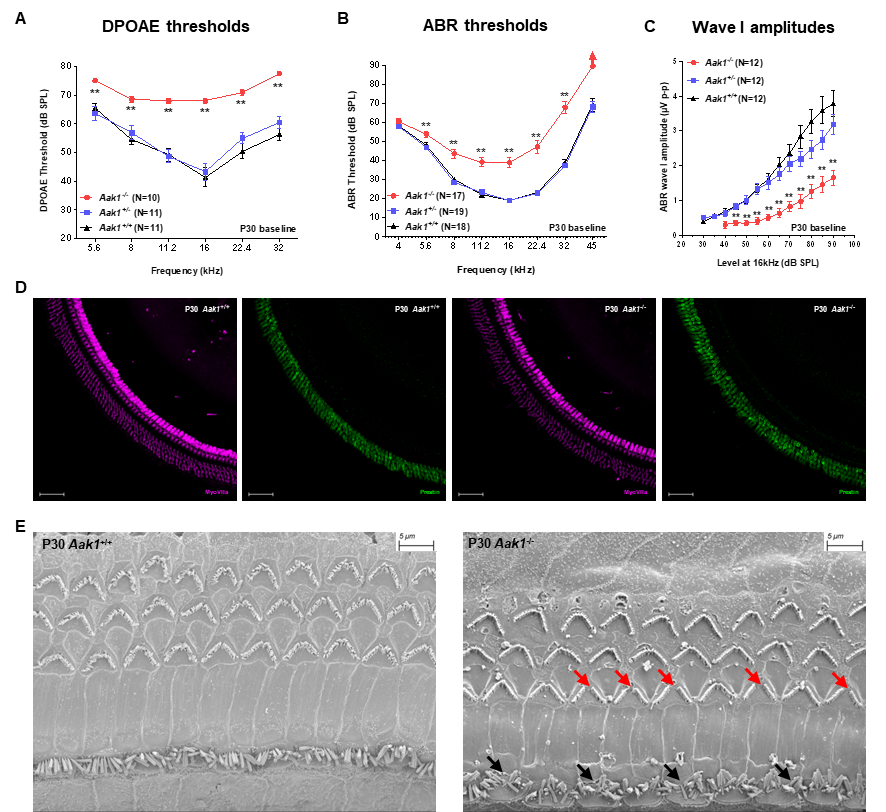


**Figure S7. Audiological characterization, cochlear immunofluorescence staining and SEM of the *Aak1* knock-out mice.** A-C show DPOAE thresholds (A), ABR thresholds (B) and wave I amplitudes (C) of the *Aak1* knock-out (*Aak1*^-/-^）mice at the age of one month (P30). The ABR wave I amplitudes were shown at the representative frequency of 16 kHz. Statistical analyses were performed using Kruskal-Wallis ANOVAs with Dunn-Bonferroni tests for post hoc comparisons (*P* values in Table S3), **P* < 0.05, ***P* < 0.01. Values and error bars reflect mean ± s.e.m. Red arrow in b indicates no ABR response at the highest stimulus level tested (90 dB SPL). (D) Whole mount cochlear immunofluorescence of middle turn showed mostly normal hair cell (marked by MyoVIIa, purple) counts and Prestin expression in the *Aak1*^-/-^ mice at P30. (E) The SEM assay showed degeneration of hair bundles of both OHCs (red arrows) and IHCs (black arrows) in the *Aak1*^-/-^ mice at P30.


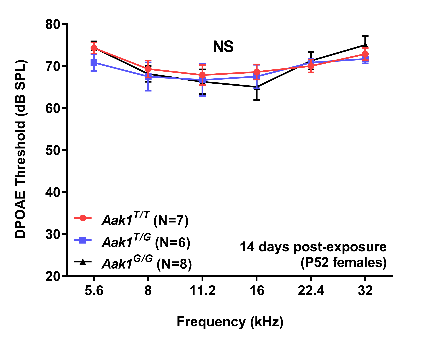

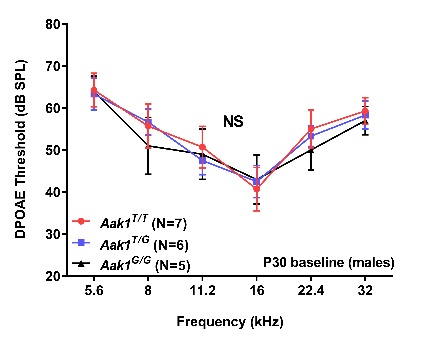

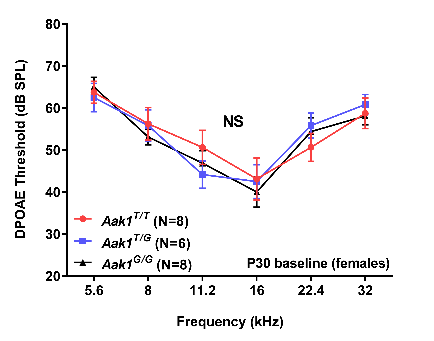

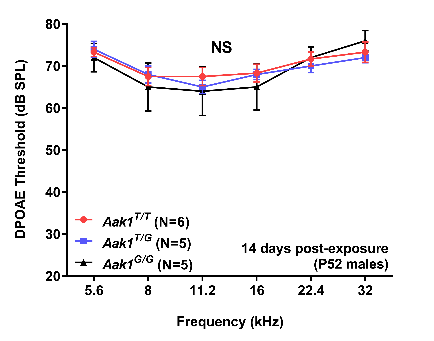

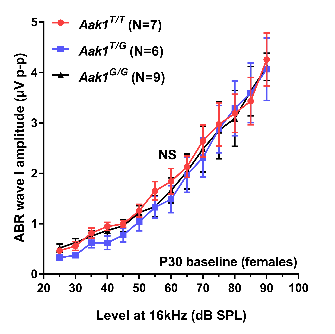

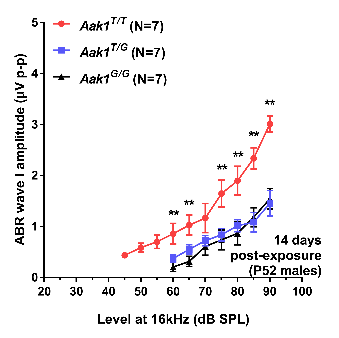

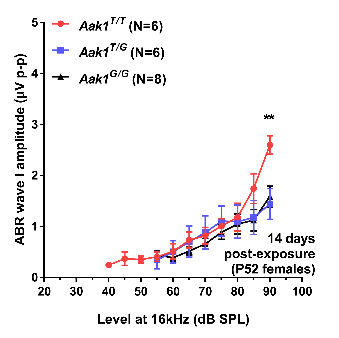

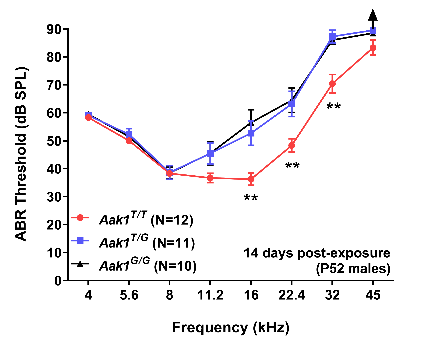

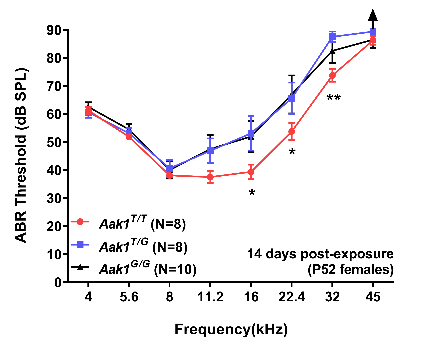

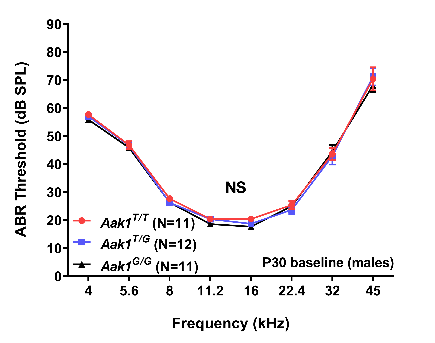

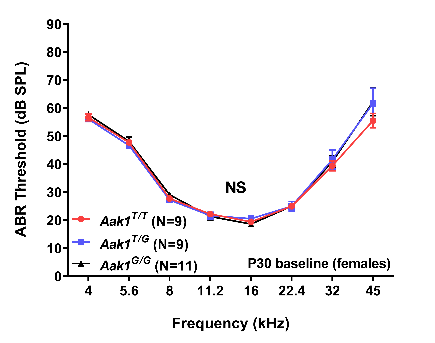

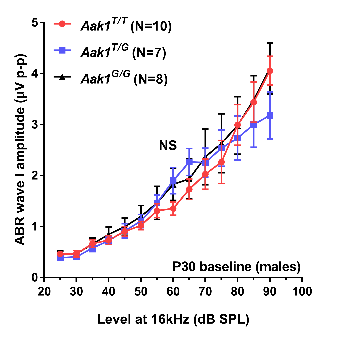
**Figure S8. Audiological characterization of the rs1396793-knock-in mice in NIHL stratified by sex.** DPOAE thresholds (A and D), ABR hearing thresholds (B and E) and wave I amplitudes (C and F, at 16kHz) of the male (upper) and female (lower) rs1396793-knock-in mice before (A-C, at P30) and 14 days after (E-F, at P52) chronic noise exposure. Black arrow in E indicates no ABR response at the highest stimulus level tested (90 dB SPL). Statistical analyses were performed using Kruskal-Wallis ANOVAs with Dunn-Bonferroni tests for post hoc comparisons (*P* values in Table S4), **P* < 0.05, ***P* < 0.01. Values and error bars reflect mean ± s.e.m.

**E**

**F**

**D**

**B**

**C**

**Females**

**Males**

**Females**

**Males**

**Wave I amplitudes**

**A**

**DPOAE thresholds**

**ABR thresholds**

**After noise exposure**

**Before noise exposure**


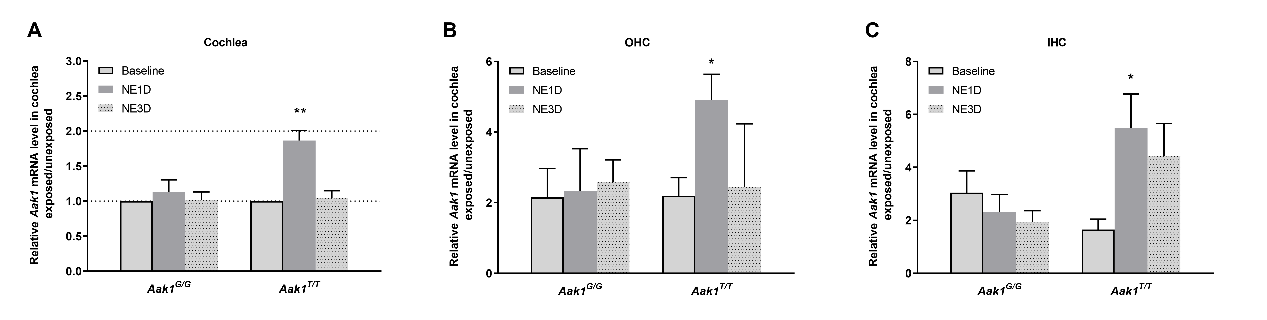


**Figure S9. Temporarily activated *Aak1* cochlear expression in the *Aak1* rs1396793-knock-in mice after noise exposure.** The fold-changes of the *Aak1* mRNA levels in cochleae of the rs1396793-knock-in mice at 1 day and 3 days after acute noise exposure (denoted as NE1D and NE3D, respectively). (A) Unlike the wild-type controls (*Aak1*^G/G^), the homozygous *Aak1*^T/T^ mice (*P* = 0.003; compared to baseline) responded to noise exposure with a temporarily activation of the *Aak1* cochlear expression (n=12 mice for each condition). Single cell RT-qPCR assays showed that *Aak1* mRNA levels were upregulated in both OHCs (B) and IHCs (C) of the homozygous *Aak1*^T/T^ mice at 1 day post-exposure (*P* = 0.028 for OHCs, *P* = 0.019 for IHCs; compared to baseline). Statistical analyses were one-sample *t* tests, **P* < 0.05, ***P* < 0.01. Values and error bars reflect mean ± s.e.m.


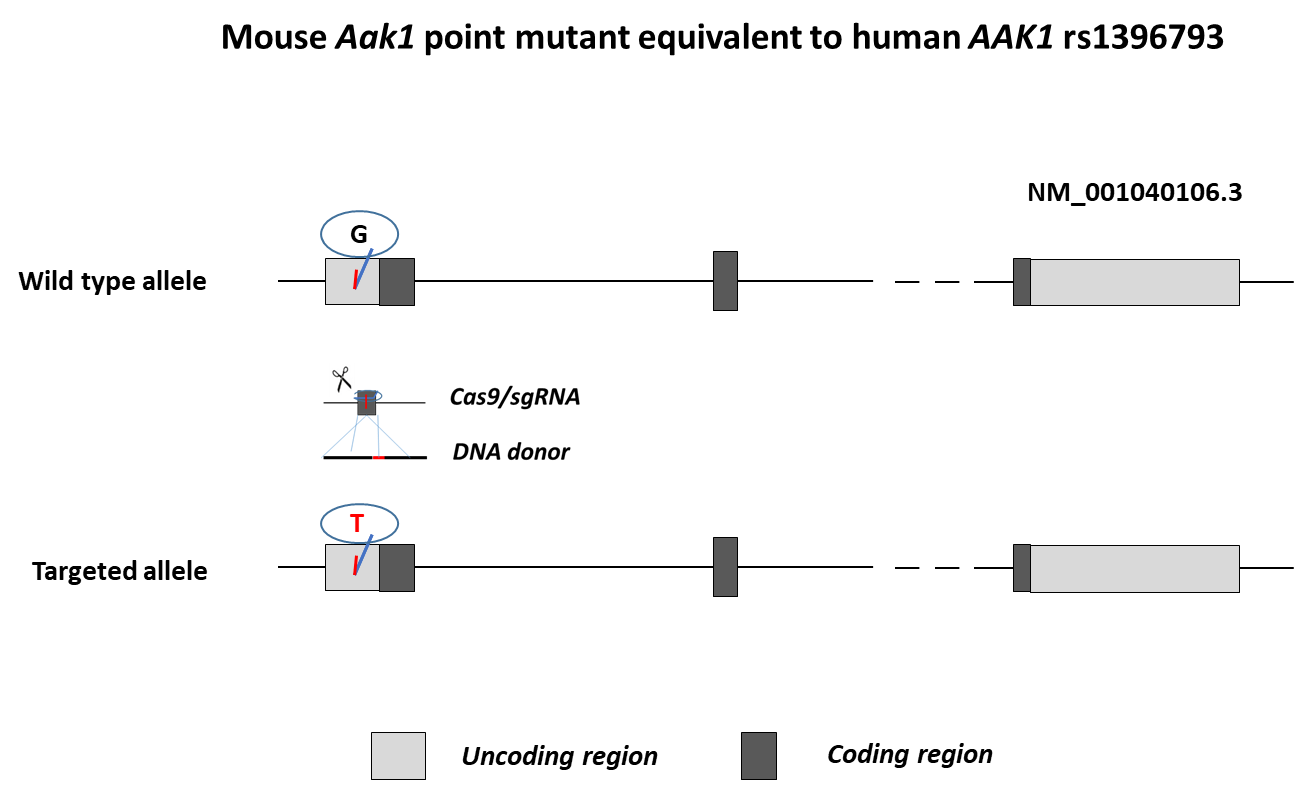

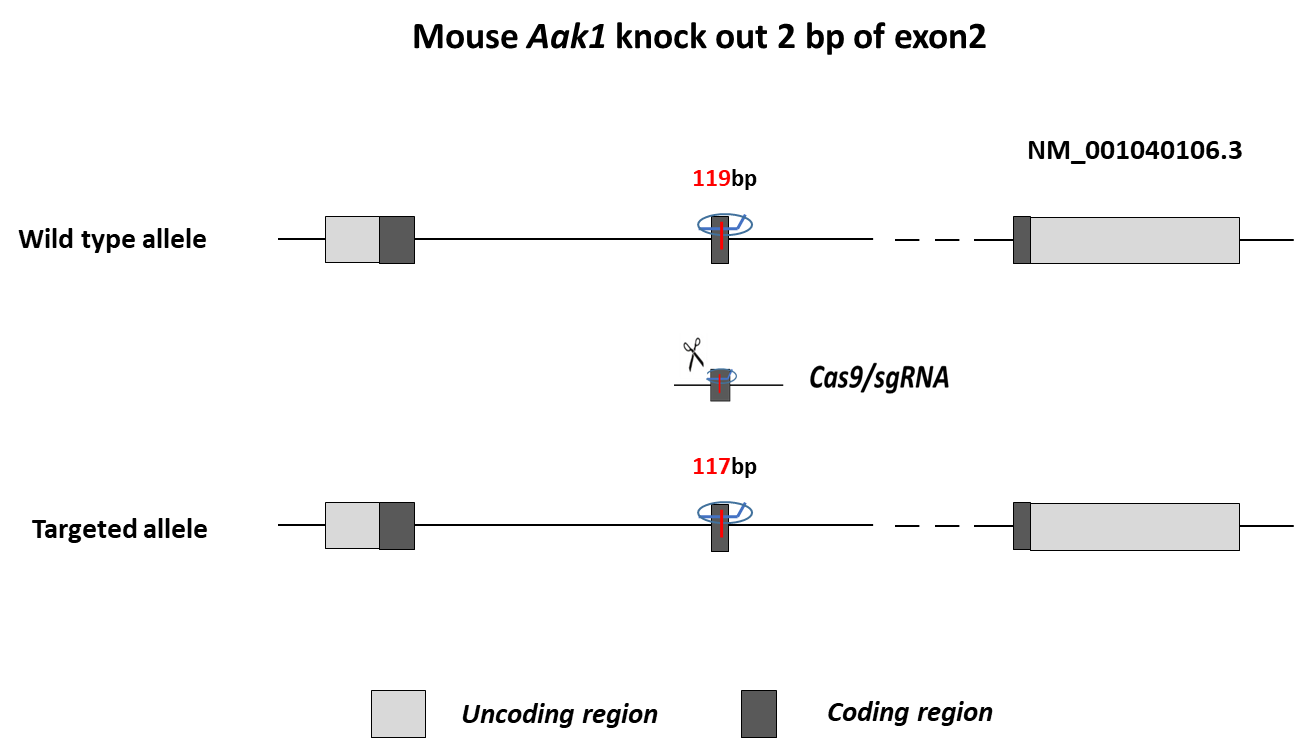

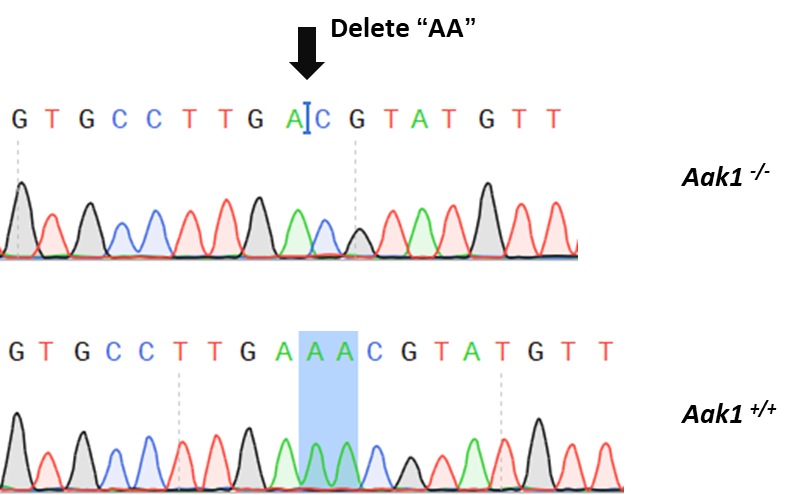

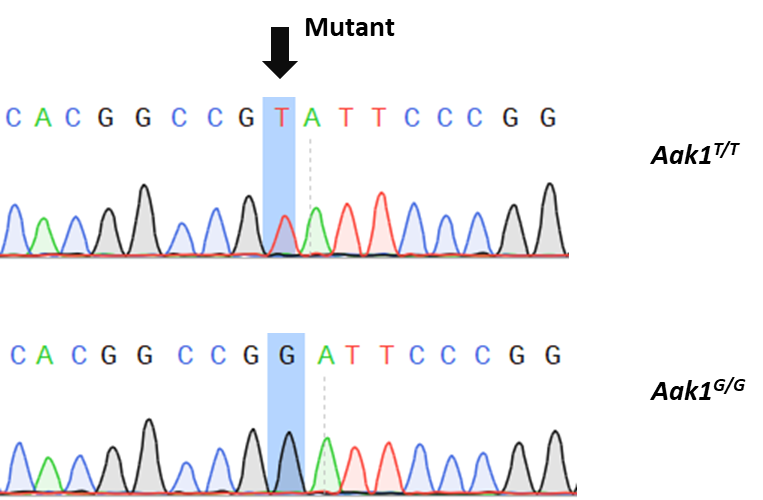
**Figure S10. Strategy for generation and genotyping of the *Aak1* knock-out and rs1396793-knock-in mice.** (A) The “G” to “T” substitution in the 5’UTR of *Aak1* (NM_001040106.3) in the mouse genome mimicking the human rs1396793 (*Aak1*^T/T^). (B) the 2-bp deletion in exon2 of *Aak1* in the knock-out (*Aak1*^-/-^) mice. Both mice models were generated via the CRISPR/Cas9 system in the C57BL/6 strain. Mice were genotyped by Sanger sequencing. (C) and (D) show the homozygous mutant and wild-type alleles in the rs1396793 knock-in and *Aak1* knock-out mice, respectively.

**A**

**B**

**C**

**D**

**SUPPLEMENTARY TABLES**

**Table S1. Characteristics of subjects in genetic studies**

| **Characteristics** | **Discovery set (n = 201)** | | **Verification set (n = 1,469)** | **Total (n = 1,670)** |
| --- | --- | --- | --- | --- |
|  | **Susceptible group (n = 101)** | **Resistant group (n = 100)** |  |  |
| **Age (years), mean (SD)** | 33.7 (5.6) | 32.5 (5.6) | 33.3 (6.9) | 33.3 (6.8) |
| **Length of working years, mean (SD)** | 5.5 (3.3) | 10.5 (4.0) | 7.4 (4.3) | 7.5 (4.3) |
| **CNE (dB[A]), mean (SD)** | 97.7 (4.3) | 101.5 (4.3) | 93.2 (5.6) | 94.0 (5.9) |
| **HPD use, n (%)** |  |  |  |  |
| **< 4 h/work-day** | 101 (100%) | 100 (100%) | 845 (57.5) | 1,046 (62.6) |
| **≥ 4 h/work-day** | 0 (0) | 0 (0) | 623 (42.4) | 623 (37.3) |
| **Earphone use, n (%)** |  |  |  |  |
| **< 1 h/day** | 101 (100%) | 100 (100%) | 1,051 (71.5) | 1,252 (75.0) |
| **≥ 1 h/day** | 0 (0) | 0 (0) | 417 (28.4) | 417 (25.0) |
| **Tobacco consumption, n (%)** |  |  |  |  |
| **< 10 cigarettes/day** | 101 (100%) | 100 (100%) | 696 (47.4) | 897 (53.7) |
| **≥ 10 cigarettes/day** | 0 (0) | 0 (0) | 772 (52.6) | 772 (46.2) |
| **Alcohol consumption, n (%)** |  |  |  |  |
| **< 50 g/day** | 101 (100%) | 100 (100%) | 1081 (73.6) | 1,282 (76.8) |
| **≥ 50 g/day** | 0 (0) | 0 (0) | 387 (26.3) | 387 (23.2) |
| **Low-frequency hearing thresholds (dB HL), mean (SD)** | 17.8 (5.9) | 13.3 (4.0) | 16.7 (5.8) | 16.5 (5.7) |
| **High-frequency hearing thresholds (dB HL), mean (SD)** | 52.0 (10.4) | 12.8 (3.6) | 26.8 (14.2) | 27.5 (15.3) |
| ***AAK1* rs1396793 (TT/TG/GG)** | (2/44/55) | (55/31/14) | (217/718/534) | (274/793/603) |

SD: standard deviation; CNE: cumulative noise exposure; HPD: hearing protection device.

**Table S2. Characteristics of subjects with different *AAK1* rs1396793 genotypes**

| **Characteristics** | **Discovery set (n = 201)** | | | **Verification set (n = 1,469)** | | |
| --- | --- | --- | --- | --- | --- | --- |
|  | T/T (n = 57) | T/G (n = 75) | G/G (n = 69) | T/T (n = 217) | T/G (n = 718) | G/G (n = 534) |
| **Age (years), mean (SD)** | 32.7 (5.6) | 32.6 (5.7) | 34.0 (5.5) | 32.5 (6.5) | 33.7 (7.0) | 33.2 (6.9) |
| **Length of working years, mean (SD)** | 10.0 (4.0) | 7.4 (4.5) | 7.0 (4.1) | 7.3 (4.1) | 7.6 (4.4) | 7.2 (4.1) |
| **CNE (dB[A]), mean (SD)** | 101.0 (3.8) | 99.2 (4.8) | 98.9 (5.0) | 94.3 (5.9) | 93.1 (5.6) | 93.0 (5.5) |
| **HPD use, n (%)** |  |  |  |  |  |  |
| **< 4 h/work-day** | 57 (100%) | 75 (100%) | 69 (100%) | 128 (59.0) | 422 (58.8) | 295 (55.2) |
| **≥ 4 h/work-day** | 0 (0) | 0 (0) | 0 (0) | 88 (40.6) | 296 (41.2) | 239 (44.8) |
| **Earphone use, n (%)** |  |  |  |  |  |  |
| **< 1 h/day** | 57 (100%) | 75 (100%) | 69 (100%) | 148 (68.2) | 519 (72.3) | 384 (71.9) |
| **≥ 1 h/day** | 0 (0) | 0 (0) | 0 (0) | 68 (31.3) | 199 (27.7) | 150 (28.1) |
| **Tobacco consumption, n (%)** |  |  |  |  |  |  |
| **< 10 cigarettes/day** | 57 (100%) | 75 (100%) | 69 (100%) | 117 (53.9) | 337 (46.9) | 242 (45.3) |
| **≥ 10 cigarettes/day** | 0 (0) | 0 (0) | 0 (0) | 99 (45.6) | 381 (53.1) | 292 (54.7) |
| **Alcohol consumption, n (%)** |  |  |  |  |  |  |
| **< 50 g/day** | 57 (100%) | 75 (100%) | 69 (100%) | 156 (71.9) | 531 (74.0) | 394 (73.8) |
| **≥ 50 g/day** | 0 (0) | 0 (0) | 0 (0) | 60 (27.6) | 187 (26.0) | 140 (26.2) |
| **Low-frequency hearing thresholds (dB HL), mean (SD)** | 13.3 (3.7) | 16.3 (5.0) | 16.6 (6.6) | 16.7 (5.2) | 16.5 (5.7) | 16.9 (6.0) |
| **High-frequency hearing thresholds (dB HL), mean (SD)** | 13.8 (7.8) | 35.7 (20.0) | 44.5 (19.5) | 24.2 (11.1) | 27.3 (14.2) | 27.2 (15.1) |

SD: standard deviation; CNE: cumulative noise exposure; HPD: hearing protection device.

**Table S3. Kruskal-Wallis ANOVA analyses on DPOAE thresholds, ABR thresholds and wave I amplitudes of the *Aak1* knock-out mice at age one month**

| **Genotypes** | **DPOAE thresholds** | | **ABR thresholds** | | **ABR wave I amplitudes** | |
| --- | --- | --- | --- | --- | --- | --- |
|  | **Frequency** | ***P* value** | **Frequency** | ***P* value** | **dB SPL at 16kHz** | ***P* value** |
| ***Aak1*^-/-^ vs. *Aak1*^+/-^** | / | / | 4 kHz | 0.290 | 45 | 0.001 |
|  | 5.6 kHz | 0.001 | 5.6 kHz | 0.004 | 50 | 0.001 |
|  | 8 kHz | 0.004 | 8 kHz | <0.001 | 55 | <0.001 |
|  | 11.2 kHz | <0.001 | 11.2 kHz | 0.001 | 60 | 0.001 |
|  | 16 kHz | 0.001 | 16 kHz | <0.001 | 65 | 0.002 |
|  | 22.4 kHz | 0.001 | 22.4 kHz | <0.001 | 70 | 0.001 |
|  | 32 kHz | 0.001 | 32 kHz | <0.001 | 75 | 0.005 |
|  | / | / | 45 kHz | <0.001 | 80 | 0.013 |
|  | / | / | / | **/** | 85 | 0.015 |
|  | / | / | / | **/** | 90 | 0.002 |
| ***Aak1*^-/-^ vs. *Aak1*^+/+^** | / | / | 4 kHz | 0.504 | 45 | 0.001 |
|  | 5.6 kHz | 0.002 | 5.6 kHz | 0.017 | 50 | <0.001 |
|  | 8 kHz | 0.001 | 8 kHz | 0.001 | 55 | <0.001 |
|  | 11.2 kHz | <0.001 | 11.2 kHz | <0.001 | 60 | <0.001 |
|  | 16 kHz | <0.001 | 16 kHz | <0.001 | 65 | <0.001 |
|  | 22.4 kHz | <0.001 | 22.4 kHz | <0.001 | 70 | <0.001 |
|  | 32 kHz | <0.001 | 32 kHz | <0.001 | 75 | <0.001 |
|  | / | / | 45 kHz | <0.001 | 80 | <0.001 |
|  | / | / | / | **/** | 85 | <0.001 |
|  | / | / | / | **/** | 90 | <0.001 |
| ***Aak1*^+/-^ vs. *Aak1*^+/+^** | / | / | 4 kHz | 1.000 | 45 | 1.000 |
|  | 5.6 kHz | 1.000 | 5.6 kHz | 1.000 | 50 | 1.000 |
|  | 8 kHz | 1.000 | 8 kHz | 1.000 | 55 | 1.000 |
|  | 11.2 kHz | 1.000 | 11.2 kHz | 1.000 | 60 | 1.000 |
|  | 16 kHz | 1.000 | 16 kHz | 1.000 | 65 | 1.000 |
|  | 22.4 kHz | 1.000 | 22.4 kHz | 1.000 | 70 | 1.000 |
|  | 32 kHz | 1.000 | 32 kHz | 1.000 | 75 | 0.245 |
|  | / | / | 45 kHz | 1.000 | 80 | 0.145 |
|  | / | / | / | **/** | 85 | 0.157 |
|  | / | / | / | **/** | 90 | 0.489 |

Multiple comparisons were performed using the Dunn-Bonferroni test. Variables without statistical significance in comparisons are not shown in this table.ABR wave I amplitudes are shown at the representative frequency of 16 kHz.

**Table S4. Kruskal-Wallis ANOVA analyses on ABR thresholds and wave I amplitudes of the rs1396793-knock-in mice 14 days after noise exposure**

| **Genotypes** | **ABR thresholds** | | | | **ABR wave I amplitudes at 16kHz** | | | |
| --- | --- | --- | --- | --- | --- | --- | --- | --- |
|  | **Frequency** | ***P* value** | | | **dB SPL** | ***P* value** | | |
|  |  | **Total** | **Males** | **Females** |  | **Total** | **Males** | **Females** |
| ***Aak1*^T/T^ vs. *Aak1*^T/G^** | 11.2 kHz | 0.035 | / | **/** | 60 | 0.239 | 0.061 | / |
|  | 16 kHz | 0.002 | 0.016 | 0.033 | 65 | 0.337 | 0.079 | **/** |
|  | 22.4 kHz | 0.018 | 0.025 | 0.082 | 70 | / | 0.416 | **/** |
|  | 32 kHz | <0.001 | <0.001 | 0.007 | 75 | 0.221 | 0.033 | **/** |
|  | 45 kHz | 0.012 | / | **/** | 80 | 0.141 | 0.033 | **/** |
|  | / | **/** | / | **/** | 85 | 0.001 | 0.001 | **/** |
|  | / | **/** | / | **/** | 90 | <0.001 | <0.001 | 0.011 |
| ***Aak1*^T/T^ vs. *Aak1*^G/G^** | 11.2 kHz | 0.036 | / | **/** | 60 | 0.025 | 0.009 | / |
|  | 16 kHz | 0.001 | 0.006 | 0.041 | 65 | 0.018 | 0.006 | **/** |
|  | 22.4 kHz | 0.006 | 0.016 | 0.029 | 70 | / | 0.203 | **/** |
|  | 32 kHz | <0.001 | 0.014 | 0.042 | 75 | 0.039 | 0.015 | **/** |
|  | 45 kHz | 0.093 | / | / | 80 | 0.051 | 0.011 | **/** |
|  | / | **/** | / | **/** | 85 | 0.001 | 0.002 | **/** |
|  | / | **/** | / | **/** | 90 | <0.001 | <0.001 | 0.018 |
| ***Aak1*^T/G^ vs. *Aak1*^G/G^** | 11.2 kHz | 1.000 | / | **/** | 60 | 1.000 | 1.000 | / |
|  | 16 kHz | 1.000 | 1.000 | 1.000 | 65 | 0.678 | 0.732 | **/** |
|  | 22.4 kHz | 1.000 | 1.000 | 1.000 | 70 | / | 1.0001 | **/** |
|  | 32 kHz | 1.000 | 1.000 | 1.000 | 75 | 1.000 | 1.000 | **/** |
|  | 45 kHz | 1.000 | / | **/** | 80 | 1.000 | 1.000 | **/** |
|  | / | **/** | / | **/** | 85 | 1.000 | 1.000 | **/** |
|  | / | **/** | / | **/** | 90 | 1.000 | 1.000 | 1.000 |

Multiple comparisons were performed using the Dunn-Bonferroni test. Variables without statistical significance in comparisons are not shown in this table.

**Table S5. The noise exposure level in six types of representative working environment in the shipyard**

| **Representative type of work** | **L_Aeq,8h_ in working environment (dB[A])** | |
| --- | --- | --- |
|  | **Mean** | **SD** |
| **Total** | 89.44 | 7.82 |
| **Grinding worker** | 102.75 | 0.85 |
| **Welder** | 93.80 | 0.40 |
| **Craneman** | 89.20 | 1.70 |
| **Assembler** | 86.80 | 0.90 |
| **Painting worker** | 84.15 | 2.85 |
| **Electrician** | 79.95 | 0.85 |

L_Aeq,8h_: equivalent A-weighted sound pressure level of continuous 8 hours; SD: standard deviation.

**Table S6. Sequences of primers and oligonucleotides used in this study.**

| **Primers/oligonucleotides** | **5’-sequence-3’** | **Usage** |
| --- | --- | --- |
| *AAK1* rs1396793 - Forward | 5’-GAATAGGGAGCAGCAAAGC-3’ | PCR amplification and sequencing for validation |
| *AAK1* rs1396793 - Reverse | 5’-GAGCCGTCTCAAGTGGGT-3’ |  |
| rs1396793 knock-in mouse sgRNA | 5’- CCGGATTCCCGGGGCTCTGCTCA -3’ | Generation of the *Aak1* knock-out and rs1396793-knock-in mice |
| rs1396793 knock-in mouse DNA donor | 5’-GATTGACAGTCTATTCCTGCAGGCGCCGTCCCTCAGCCCCCACCCCACGGCCGTATTCCCGGGGCTCTGCTCAGGGCTGAAACCGGAGAAGAAATCCTTAGATCTC -3’ |  |
| *Aak1* knock-out mouse sgRNA | 5’- CCTTGAAACGTATGTTTGTCAAC -3’ |  |
| rs1396793 knock-in mouse-Forward | 5’- TAGGCTCTGGAGTGTGTTACTG -3’ | PCR amplification and sequencing of *Aak1* in mice |
| rs1396793 knock-in mouse- Reverse | 5’ - AAGACCCTTCCGATGTAACCAC -3’ |  |
| *Aak1* knock-out mouse-Forward | 5’ - GTGCCAACAGTAGCCAGAGTTC -3’ |  |
| *Aak1* knock-out mouse-Reverse | 5’ - AAGAAGAAGCCACAGTCACAATG -3’ |  |
| Mouse *Aak1* qPCR-Forward | 5’- CGCTCTTGTCTTTCTGGTGA-3’ | Real-time quantitative RT-PCR of mouse cochlea |
| Mouse *Aak1* qPCR- Reverse | 5’- TGTTCTTGTGCCCTGATAGGT-3’ |  |
| Mouse β-actin qPCR-Forward | 5’- AAGATCAAGATCATTGCTCCTCC-3’ |  |
| Mouse β-actin qPCR- Reverse | 5’- GACTCATCGTACTCCTGCTTGC-3’ |  |
| Mouse *Otof* qPCR-Forward | 5’-CTGACACGGCATTCGTCTG-3’ |  |
| Mouse *Otof* qPCR- Reverse | 5’-CCTGGGAGGCTGTAAAGGAA-3’ |  |
| Mouse *Slc26a4* qPCR-Forward | 5’-CACTCATTATGGGAGCGAGA-3’ |  |
| Mouse *Slc26a4* qPCR- Reverse | 5’-TCCGTCTACTTCTGCATCCAC-3’ |  |

**REFERENCES**

1. Zhao YM, Qiu W, Zeng L *et al.* Application of the kurtosis statistic to the evaluation of the risk of hearing loss in workers exposed to high-level complex noise. *Ear Hear*. 2010; **31**(4): 527-532. doi: 10.1097/AUD.0b013e3181d94e68

2. Wang Q, Qian M, Yang L *et al.* Audiometric Phenotypes of Noise-Induced Hearing Loss by Data-Driven Cluster Analysis and Their Relevant Characteristics. *Front med (Lausanne)*. 2021; **8**: 662045. doi: 10.3389/fmed.2021.662045

3. Milon B, Mitra S, Song Y *et al.* The impact of biological sex on the response to noise and otoprotective therapies against acoustic injury in mice. *Biology of sex differences*. 2018; **9**(1): 12. doi: 10.1186/s13293-018-0171-0

4. Wang Q, Wang X, Yang L *et al.* Sex differences in noise-induced hearing loss: a cross-sectional study in China. *Biology of sex differences*. 2021; **12**(1): 24. doi: 10.1186/s13293-021-00369-0

5. Kujawa SG, Liberman MC. Translating animal models to human therapeutics in noise-induced and age-related hearing loss. *Hearing research*. 2019; **377**: 44-52. doi: 10.1016/j.heares.2019.03.003

6. Carroll YI, Eichwald J, Scinicariello F *et al.* Vital Signs: Noise-Induced Hearing Loss Among Adults - United States 2011-2012. *MMWR Morbidity and mortality weekly report*. 2017; **66**(5): 139-144. doi: 10.15585/mmwr.mm6605e3

7. McKenna A, Hanna M, Banks E *et al.* The Genome Analysis Toolkit: a MapReduce framework for analyzing next-generation DNA sequencing data. *Genome research*. 2010; **20**(9): 1297-1303. doi: 10.1101/gr.107524.110

8. DePristo MA, Banks E, Poplin R *et al.* A framework for variation discovery and genotyping using next-generation DNA sequencing data. *Nature genetics*. 2011; **43**(5): 491-498. doi: 10.1038/ng.806

9. Li H, Durbin R. Fast and accurate short read alignment with Burrows-Wheeler transform. *Bioinformatics (Oxford, England)*. 2009; **25**(14): 1754-1760. doi: 10.1093/bioinformatics/btp324

10. Firtina C, Alkan C. On genomic repeats and reproducibility. *Bioinformatics (Oxford, England)*. 2016; **32**(15): 2243-2247. doi: 10.1093/bioinformatics/btw139

11. Wang K, Li M, Hakonarson H. ANNOVAR: functional annotation of genetic variants from high-throughput sequencing data. *Nucleic acids research*. 2010; **38**(16): e164. doi: 10.1093/nar/gkq603

12. Karczewski KJ, Francioli LC, Tiao G *et al.* The mutational constraint spectrum quantified from variation in 141,456 humans. *Nature*. 2020; **581**(7809): 434-443. doi: 10.1038/s41586-020-2308-7

13. Anderson CA, Pettersson FH, Clarke GM *et al.* Data quality control in genetic case-control association studies. *Nature protocols*. 2010; **5**(9): 1564-1573. doi: 10.1038/nprot.2010.116

14. Chang CC, Chow CC, Tellier LC *et al.* Second-generation PLINK: rising to the challenge of larger and richer datasets. *Gigascience*. 2015; **4**: 7-7. doi: 10.1186/s13742-015-0047-8

15. Moore JE, Purcaro MJ, Pratt HE *et al.* Expanded encyclopaedias of DNA elements in the human and mouse genomes. *Nature*. 2020; **583**(7818): 699-710. doi: 10.1038/s41586-020-2493-4

16. Cong L, Ran FA, Cox D *et al.* Multiplex genome engineering using CRISPR/Cas systems. *Science (New York, NY)*. 2013; **339**(6121): 819-823. doi: 10.1126/science.1231143

17. Qian M, Wang Q, Wang Z *et al.* Dose-Dependent Pattern of Cochlear Synaptic Degeneration in C57BL/6J Mice Induced by Repeated Noise Exposure. *Neural plasticity*. 2021; **2021**: 9919977. doi: 10.1155/2021/9919977

18. Liu H, Peng H, Wang L *et al.* Differences in Calcium Clearance at Inner Hair Cell Active Zones May Underlie the Difference in Susceptibility to Noise-Induced Cochlea Synaptopathy of C57BL/6J and CBA/CaJ Mice. *Frontiers in cell and developmental biology*. 2020; **8**: 635201. doi: 10.3389/fcell.2020.635201

19. Kroll J, Jaime Tobón LM, Vogl C *et al.* Endophilin-A regulates presynaptic Ca(2+) influx and synaptic vesicle recycling in auditory hair cells. *Embo j*. 2019; **38**(5). doi: 10.15252/embj.2018100116
